# Acid-catalyzed rearrangements in arenes: interconversions in the quaterphenyl series

**DOI:** 10.3762/bjoc.15.258

**Published:** 2019-11-06

**Authors:** Sarah L Skraba-Joiner, Carter J Holt, Richard P Johnson

**Affiliations:** 1 Department of Chemistry and Materials Science Program, University of New Hampshire, Durham, NH 03824, USA

**Keywords:** arenium ion, carbocation, density functional theory, microwave reaction, rearrangement, superacid

## Abstract

Arenes undergo rearrangement of phenyl, alkyl, halogen and other groups through the intermediacy of *ipso* arenium ions in which a proton is attached at the same carbon as the migrating substituent. Interconversions among the six quaterphenyl isomers have been studied here as a model for rearrangements of linear polyphenyls. All reactions were carried out in 1 M CF_3_SO_3_H (TfOH) in dichloroethane at 150 °C in a microwave reactor for 30–60 min, with product formation assessed by high field NMR analysis. Under these reaction conditions, *m,p'*-quaterphenyl is the equilibrium product. This isomer is unchanged by the reaction conditions and all other quaterphenyl isomers rearrange to *m,p'* as the dominant or sole product. DFT computations with inclusion of implicit solvation support a complex network of phenyl and biphenyl shifts, with barriers to rearrangement in the range of 10–21 kcal/mol. Consistent with experiments, the lowest energy arenium ion located on this surface is due to protonation of *m,p'*-quaterphenyl. This supports thermodynamic control based on carbocation energies.

## Introduction

Carbocations are enigmatic reactive intermediates of enduring importance in chemistry. No other reactive species displays such a complex and fascinating collection of molecular rearrangements. Building on a long history, new synthetic applications [1–2] and explanations of carbocation reaction mechanisms [3–6] continue to be discovered. Chemistry in superacid solutions has played a major role in this field [7–8].

**Scheme 1:**
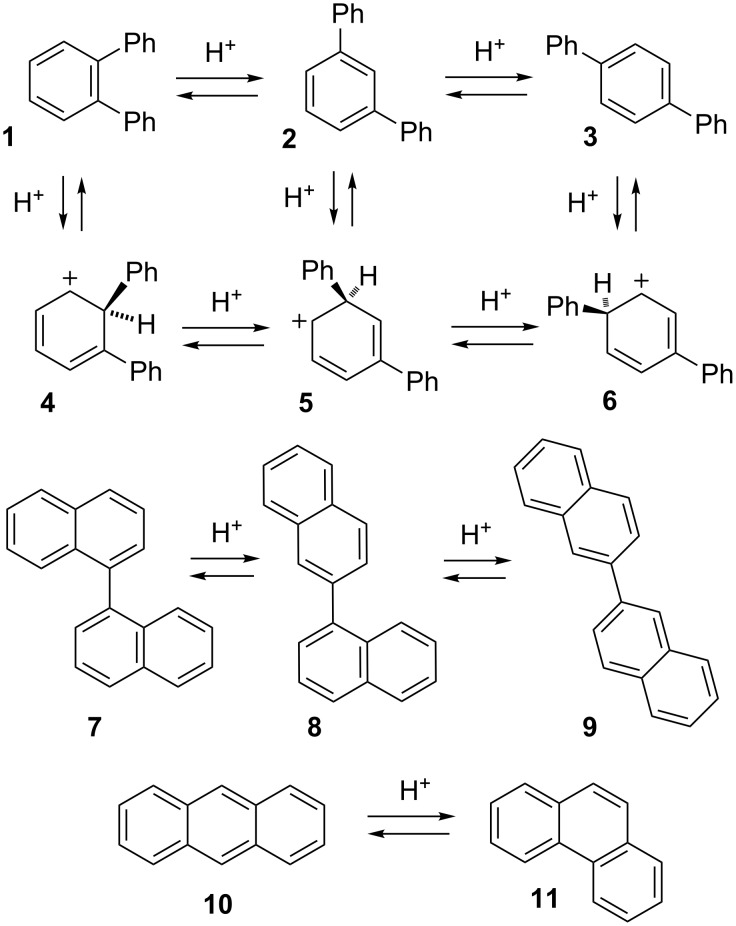
Acid-catalyzed rearrangements of arenes.

Every student of organic chemistry is taught the importance of arenium ions in the classic two step S_E_Ar mechanism for electrophilic aromatic substitution. Addition of an electrophile to an arene leads to a bound species, sometimes called a σ-complex, which then loses a proton at the site of substitution to yield the product [9]. Of course challenges to this simple mechanism exist [10–15], including the recent proposal of a one-step process [16]. Reaction dynamics of electrophile–arene π complexes may also play a role in site selectivity [17]. It is less commonly known that arenium ions, like many other types of carbocations, often rearrange by 1,2-shifts. This leads to a fascinating collection of rearrangements that can migrate hydrogen, halogens or more complex substituents around the ring and even modify the carbon skeleton. Early reports by Baddeley [18] helped to explain odd results from Friedel–Crafts reactions [19] and this type of process is sometime referred to as a Baddeley rearrangement. Many examples of alkyl group migration have been described [19]. Phenyl groups migrate easily and degenerate phenyl shifts in biphenyl were confirmed by isotopic labeling [20–21]. One classic example of arenium ion chemistry is the interconversion of terphenyl isomers **1**–**3** (Scheme 1). This rearrangement was first reported by Allen and Pingert in 1942 [22] and then independently rediscovered by Olah and Meyer twenty years later [23]. Interconversion of isomers **1**–**3** is believed to occur through the intermediacy of *ipso* arenium ions **4**–**6** which connect through 1,2-phenyl shifts.

The term "*ipso*" was first proposed by Perrin and Skinner to explain unusual results in electrophilic substitution reactions; this refers to protonation at the site of a substituent [24]. *Ipso* protonation is the essential step in arenium ion rearrangements.

Our interest in this field arose from an accidental rediscovery of rearrangements in the terphenyl series (**1**–**3**; Scheme 1), by heating **1** with AlCl_3_ – a reaction independently discovered twice before [22–23]! We confirmed earlier observations of facile acid-catalyzed interconversion, as well as the fact that m-terphenyl (**2**) is favored at equilibrium (observed ratio **1**:**2**:**3** is <1:69:31) and used theory to explain this product selectivity [25]. This preference is consistent with formation of the most stable arenium ion intermediate, as shown by DFT computations.

Early studies in this field used AlCl_3_ as catalyst and it was generally assumed that adventitious traces of water generated the actual catalyst, precise identity unknown. We found these "water-promoted" reactions with catalytic AlCl_3_ to be unreliable. To remove ambiguity about the catalyst and extend the temperature range, we developed a more reliable method for studying higher temperature carbocation rearrangements. In our method, we use 1 M (ca. 20% by volume) trifluoromethanesulfonic acid (TfOH) as catalyst with dichloroethane as our preferred solvent. Most importantly, reactions are conducted in the capped tube of a microwave reactor. With this approach, we can safely and reproducibly heat reactions to ca. 170 °C, so far without incident.

Other more complex rearrangements are easily observed. In the binaphthyl series **7**–**9**, three sequential rearrangements occur at ambient temperature favoring the 2,2'-isomer **9** (97%) at equilibrium [26]. Aryl shifts occur readily in naphthalene, with beta-substitution favored at equilibrium. Skeletal rearrangements of fused arene rings are also possible and can proceed through several mechanisms. The first example was reported by Dansi and Salvioni in 1941 in the rearrangement of benz[*a*]anthracene to chrysene [27]. We recently studied the rearrangement of anthracene (**10**) to phenanthrene (**11**) [28], finding evidence to support a complex process, suggested earlier [29], that involves initial reduction to 1,2,3,4-tetrahydroanthracene, followed by a pirouette rearrangement of the reduced ring through a spirocyclic intermediate and then re-oxidation to phenanthrene. These reactions involve a complex series of proton and hydride transfers.

## Results and Discussion

**Scheme 2:**
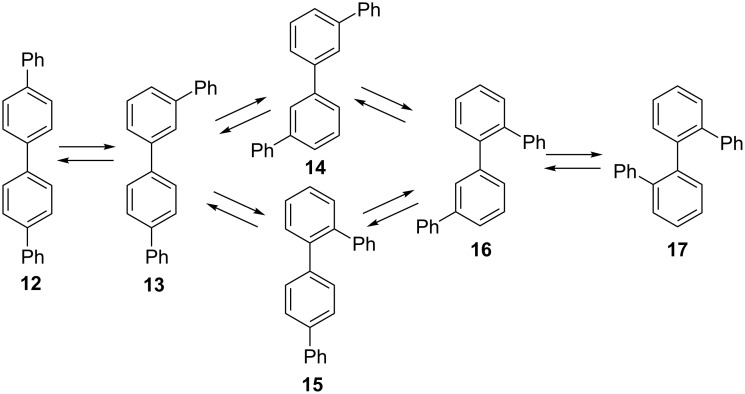
Rearrangement of quaterphenyl isomers by phenyl shifts.

Beyond terphenyls, acid-catalyzed rearrangements pose limitations in the synthesis of extended polyphenyls but the factors controlling interconversion of isomers are poorly understood [30–33]. As one recent example, Jasti and co-workers showed that cycloparaphenylenes undergo rapid acid-catalyzed rearrangement which precludes using Scholl-type chemistry in this series [30]. In the present work, we have explored rearrangements of quaterphenyls, the next homolog in the paraphenylene series, now with six structural isomers. Scheme 2 summarizes interconnections via 1,2-migration of a terminal phenyl group. As will be shown below, an internal aryl–aryl bond can also be transposed through 1,2-biphenyl migration, with a similar network of interconversions. Based on the behavior of terphenyl which favors *meta* substitution at equilibrium, we initially hypothesized that *m,m'-*quaterphenyl (**14**) would likely be the major isomer at equilibrium.

Only brief exploration of quaterphenyl rearrangements had been described previously. Isomerization of *o*,*o*’-quaterphenyl (**17**) with SnCl_4_/AlCl_3_ catalysis has been reported to yield a mixture of *p*,*p*’- (**12**), *m*,*p*’- (**13**), and *m*,*m*’-quaterphenyl (**14**) [34].

**Scheme 3:**
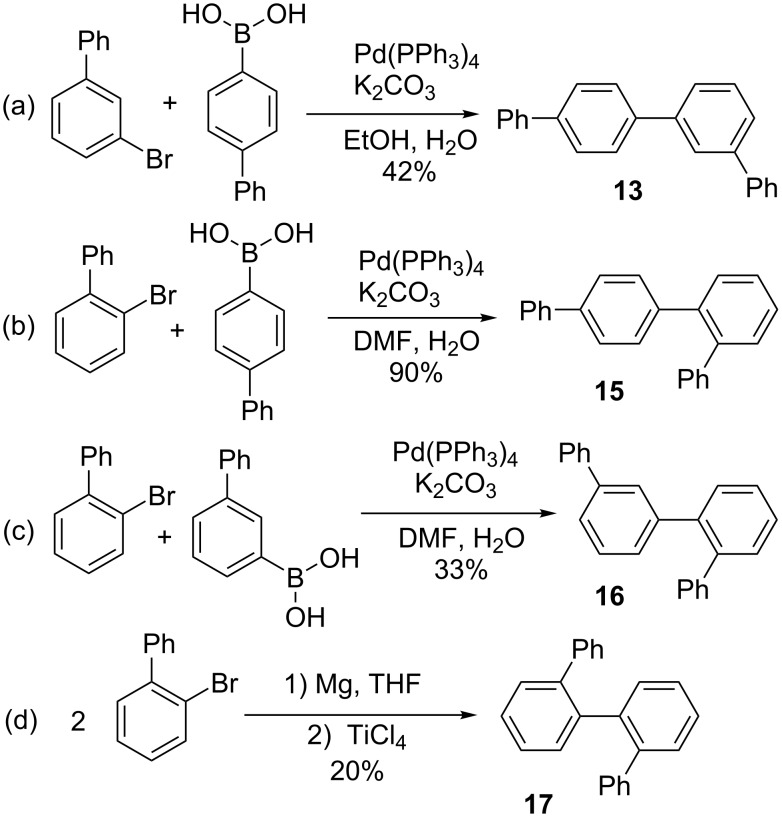
Synthesis of quaterphenyl isomers.

*p*,*p*’-Quaterphenyl (**12**) and *m*,*m*’-quaterphenyl (**14**) were available commercially. To complete the series, samples of *m*,*p*’- (**13**), *o*,*p*’- (**15**), *o*,*m*’- (**16**), and *o*,*o*’-quaterphenyl (**17**) were synthesized as shown in Scheme 3. Suzuki–Miyaura coupling was used to synthesize **13**, **15**, and **16** from the corresponding aryl bromides and boronic acids [35]. *o*,*o*’-Quaterphenyl (**17**) was synthesized by homo-coupling of 2-bromobiphenyl, as previously reported [36].

Promoting the rearrangement of all quaterphenyl isomers at room temperature or in refluxing DCE (84 °C) proved to be difficult because *p*,*p*’-quaterphenyl (**12**) is very poorly soluble in this solvent and higher temperatures were required in some cases to reach equilibrium. In a solution of 1 M TfOH/DCE, **12** forms a colored solution more slowly (ca. 1–2 min) than previous examples we have studied. Heating these reaction mixtures in a microwave reactor allows for the substrate to dissolve in the reaction medium, resulting in a rearrangement. To provide a consistent reaction environment, the rearrangement of all isomers was studied using a microwave reactor at 150 °C, with reaction times of 30 or 60 min. After chromatographic purification to remove oligomeric material, product distributions were determined by high field ^1^H NMR, with integration of unique resonances for each isomer. In these reactions, we attribute no special effect due to microwaves. This has been a subject of some debate in the literature [37–38]. As we have demonstrated earlier, the microwave reactor simply provides a safe and reliable way to heat samples with superacids at temperatures well above the normal solvent boiling point.

The results of quaterphenyl isomerizations are summarized in Table 1. Initially it was expected that *m*,*m*’-quaterphenyl (**14**) might be the major isomer formed at equilibrium, as previous examples have heavily favored *meta-*substitution patterns. Contrary to this expection, the major product observed in all cases was *m*,*p*’-quaterphenyl (**13**). In 30 min at 150 °C, *p*,*p*’-quaterphenyl (**12**) isomerized completely to **13**. Under the same conditions, *m*,*m*’-quaterphenyl (**14**) isomerized more slowly, yielding **13** and **14** in a 50:50 mixture in 30 min. Increased reaction time with *m,m*’-quaterphenyl (**14**) yielded **13** and **14** in a 67:33 ratio. Starting with *o*,*m*’- (**16**) or *o*,*o*’-quaterphenyl (**17**) yielded a mixture of ca. 60% **13** and ca. 40% **14** in 30 min. Similarly, *o*,*p*’-quaterphenyl (**15**) isomerized to an 81:19 mixture of **13**:**14**. When starting with *m*,*p*’-quaterphenyl, no rearrangement to **14** was observed, even at increased reaction times.

**Table 1:** Product distributions from rearrangement of quaterphenyl isomers.^a,b^.

Reactant	Time (min)	**12**	**13**	**14**	**15**	**16**	**17**	Yield

**12**	30	–	100	–	–	–	–	70
**12**	60	–	100	–	–	–	–	55
**13**	30	–	100	–	–	–	–	88
**13**	60	–	100	–	–	–	–	75
**14**	30	–	50	50	–	–	–	81
**14**	60	–	67	33	–	–	–	90
**15**	30	–	81	19	–	–	–	70
**16**	30	–	57	43	–	–	–	64
**17**	30	–	60	40	–	–	–	67

^a^All reactions were carried out in 1 M CF_3_SO_3_H in dichloroethane at 150 °C in a microwave reactor. ^b^Yields are total isolated products after flash chromatography.

Minor products with more downfield chemical shifts were also observed *via* ^1^H NMR of these crude product mixtures; these were easily separated from quaterphenyl isomers using flash column chromatography. Analysis of the crude product mixtures by MALDI–TOF–MS confirmed these minor products could be attributed to oligomerization (*m/z* = 344, 673).

Our conclusion from this series of experiments is that *m,p'*-quaterphenyl (**13**) is the equilibrium product from rearrangement of all of the six isomers. This is most clearly demonstrated in the rearrangement of **12** to **13** and the failure of **13** to produce other isomers. In some cases, reaction times were insufficient to fully reach equilibrium.

## Computational models for quaterphenyl rearrangements

**Scheme 4:**
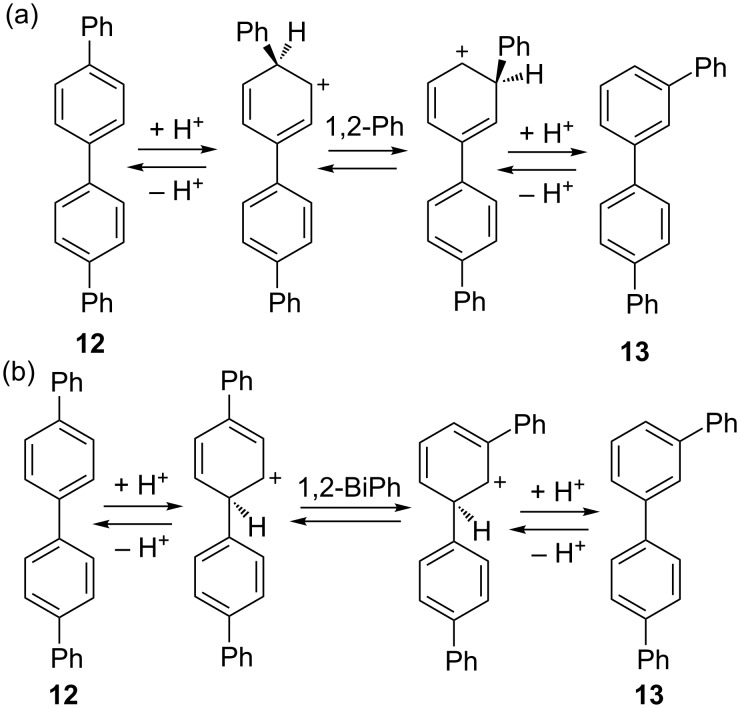
Rearrangement of quaterphenyl isomers via (a) 1,2-phenyl shift and (b) 1,2-biphenyl shift.

While the energy surface for the interconversion of quaterphenyl isomers initially seems straightforward, there are additional modes of rearrangement that must be considered. 1,2-Phenyl shifts are possible to interconvert isomers, occurring through protonation at the *ipso* site on the external phenyl rings (Scheme 4a). Biphenyl shifts are also possible through protonation at the internal *ipso* sites (Scheme 4b). This results in a very complex potential energy surface. Protonation of a terminal phenyl group can also lead to terphenyl migration but this process is structurally degenerate and was not explored by computations.

Both phenyl and biphenyl rearrangement pathways were studied by DFT methods, with inclusion of implicit solvation by the polarizable continuum model (PCM). As in our earlier research, we employed B3LYP/6-31+G(d,p) theory with PCM solvation in dichloroethane [25–26,28]. As noted earlier by Tantillo, the B3LYP functional provides a very good description of carbocation chemistry [3].

**Figure 1:**
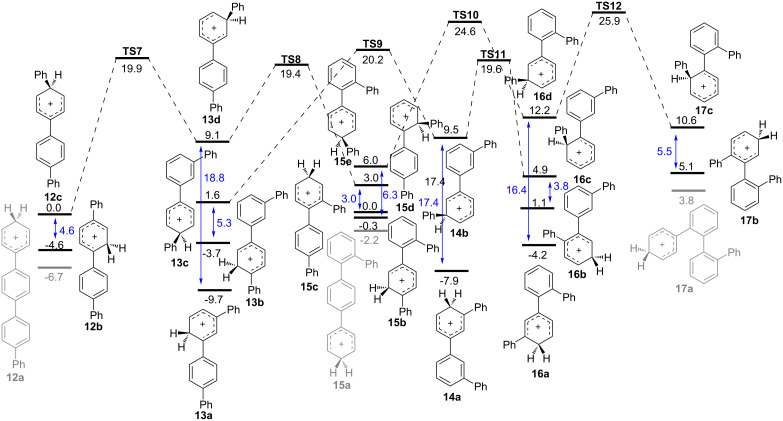
Pathways for terminal 1,2-phenyl shifts in quaterphenyl isomers calculated with IEFPCM(DCE)/B3LYP/6-31+G(d,p) theory. Relative free energies are given in kcal/mol.

**Figure 2:**
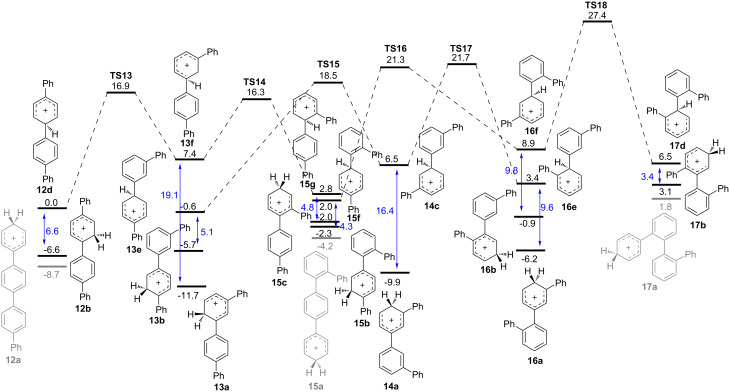
Pathways for 1,2-biphenyl shifts in quaterphenyl isomers calculated with IEFPCM(DCE)/B3LYP/6-31+G(d,p) theory. Relative free energies are given in kcal/mol.

Figure 1 shows the lowest energy pathways for 1,2-phenyl shifts, while Figure 2 shows those for a 1,2-biphenyl shifts. In each case, the energy reference is arbitrarily chosen as the linear *ipso* cation **12c** or **12d** for phenyl or biphenyl shift, respectively. Predicted barriers for rearrangements of *ipso* cations are all in the range of 9–22 kcal/mol. Common to both potential energy surfaces are the lowest energy non-*ipso* carbocations **12a**–**17a** which lie at the bottom on the energy scale. It is noteworthy that *m,p'* cation **13a** is the lowest energy species predicted by our calculations. In each diagram, double-headed vertical arrows show the energy difference between *ipso* cations and their non-*ipso* counterpart which might be formed rapidly by secondary 1,2-hydride shifts.

In principle, the equilibrium product in these complex reactions might correlate with relative energies of the neutral quaterphenyl isomers. Reliable heats of formation for quaterphenyls are unavailable in the literature but these values and other estimates for relative energies can be predicted by theory. Starting with the lowest energy conformer for each quaterphenyl, we first computed heats of formation using the T1 method [39]. Predicted values (Supporting Information File 1) are narrowly clustered in a range of ca. 2 kcal/mol, with the lowest energy isomer predicted to be *o*,*o*'-quaterphenyl (**17**). This low ranking for the most congested isomer may be attributed to intramolecular π stacking. Very similar results were obtained with M06-2X/6-311+G(d,p) theory, which also placed **17** as the lowest energy quaterphenyl isomer. Our results thus do not support thermodynamic control based on relative energies of the neutral quaterphenyls.

A more plausible scenario is that thermodynamic control applies to carbocation intermediates, with the equilibrium product determined by the energy of the lowest energy carbocation in solution. This is predicted to be **13a**. The speed of equilibration for different isomers is determined by the number of required steps and their barriers. Thus **12** rearranges to **13** in a phenyl or biphenyl migration, passing through **TS7** or **TS13**, respectively. This rearrangement is complete in our standard 30 min reaction time. By contrast, **17** requires three separate migration steps to arrive at **13**, passing through **16** and **14**; this rearrangement (Table 1) is incomplete during the same reaction period.

An unknown factor is the degree of protonation, especially at 150 °C. We observed earlier by NMR spectroscopy that anthracene is fully protonated at ambient temperature in 1M TfOH but 1,3,5-triphenylbenzene is not [28]. One experimental observation is that the 1M TfOH/arene reaction solutions invariably have a bright color at ambient temperature due to the carbocation [40–41]. As these solutions are heated, they become much darker in color, implying a higher level of protonation. Upon cooling, a more normal color is returned. Full protonation of **13** at 150 °C would explain thermodynamic control by cationic intermediates.

Our naïve supposition at the outset of this project that *m*,*m'*-quaterphenyl **14** might be favored at equilibrium was unsupported by experiment, which instead showed the *m*,*p'* isomer **13** to be preferred. A comparison of **5a**, the lowest energy cation from terphenyl rearrangements, with **13a**, the corresponding cation for quaterphenyls, provides a simple explanation. If a single ring is protonated, greater stability accrues from a *para* phenyl substituent with *meta* a close second. The same effect should apply to longer polyphenylene chains, resulting in a chain of aryl groups that remains mostly *para* after generating a more basic *meta* substituted site.


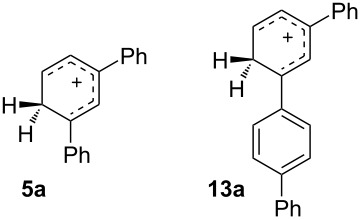


## Conclusion

Acid-catalyzed interconversions among the six quaterphenyl isomers have been studied in this work as a model for rearrangements of linear polyphenyls. All reactions were carried out in 1 M CF_3_SO_3_H (TfOH) in dichloroethane at 150 °C in a microwave reactor for 30 or 60 min, with product formation assessed by high field NMR analysis. Under these reaction conditions, *m,p'*-quaterphenyl (**13**) is the equilibrium product. This isomer is unchanged by the reaction conditions and all other quaterphenyl isomers rearrange to *m,p'* as the dominant or sole product. DFT computations with inclusion of implicit solvation support a complex network of phenyl and biphenyl shifts, with barriers to rearrangement in the range of 10–21 kcal/mol. Consistent with experiments, the lowest energy arenium ion located on this surface is due to protonation of *m,p'*-quaterphenyl. This supports thermodynamic control based on carbocation energies. The same effect may apply to longer polyphenylene chains, resulting in a chain of aryl groups that does not fully rearrange but remains mostly *para* after generating a more basic *meta* substituted site.

## Experimental

**General methods.** Trifluoromethanesulfonic acid (TfOH, 99% purity) and dichloroethane (DCE, 99+%) were used as received from commercial sources. Glassware was oven dried and all reactions were run under a nitrogen atmosphere. ^1^H NMR spectra were measured in CDCl_3_ using a Varian XL-400 MHz spectrometer. Microwave reactions were conducted using a single-mode CEM microwave reactor in 10 mL vessels with temperature monitoring by an external sensor.

**General procedure for rearrangement in the microwave (MW) reactor.** In a similar manner as described in [28], under a nitrogen atmosphere, the substrate (ca. 4 – 50 mg) and 1,2-dichloroethane (DCE, 4 mL) were added to a 10 mL reaction vessel. Trifluoromethanesulfonic acid (TfOH) (0.40 mL, 4.5 mmol) was added dropwise by syringe; this typically caused formation of a bright color. The reaction mixture was purged with nitrogen, capped and heated in a microwave reactor. Reaction times represent hold times after a ramp time of ca. 10 minutes. After cooling, products were isolated by careful neutralization with saturated aqueous NaHCO_3_ and extraction with dichloromethane. The crude product mixture was purified by flash chromatography then analyzed using ^1^H NMR. The presence of oligomeric material was assessed on crude isolated product through time-of-flight matrix assisted laser desorption ionization (MALDI–TOF–MS) mass spectrometry, using sulfur as a matrix.

**Suzuki–Miyaura coupling to form *m*,*p*’-quaterphenyl (13)** [35]**:** 4-Biphenylboronic acid (0.18 g, 0.91 mmol) and 1 M K_2_CO_3_ (1.5 mL) were added to a 10 mL Pyrex microwave tube. 3-Bromobiphenyl (0.07 mL, 0.42 mmol), ethanol (2.3 mL), and Pd(PPh_3_)_4_ (28 mg, 0.24 mmol) were then added. The head-space was purged with nitrogen and the tube was capped before the reaction mixture was placed in a microwave reactor (150 °C, 30 min hold time). After the reaction, the mixture was quenched with 1 M NaOH, extracted with dichloromethane, washed with water and brine, and dried with Na_2_SO_4_. The organic layer was then concentrated to a brown solid and purified by chromatography with hexanes to yield *m,p’*-quaterphenyl (**13**) as a white solid (0.058 g, mp 163–166 °C, lit 167–168 °C, 42% yield); ^1^H NMR (400 MHz, CDCl_3_) δ 7.87–7.85 (m, 1H), 7.76–7.69 (m, 4H), 7.69–7.64 (m, 4H), 7.64–7.58 (m, 2H), 7.57–7.52 (m, 1H), 7.50–7.45 (m, 4H), 7.41–7.35 (m, 2H).

**Suzuki–Miyaura coupling to form o,*p*’-quaterphenyl (15)** [35]**:** K_2_CO_3_ (0.91 g, 6.6 mmol) and water (7.7 mL) were combined in a 25 mL round bottom flask which was purged with nitrogen, and cooled to 0 °C. Biphenyl-4-boronic acid (0.89 g, 4.5 mmol) was added, followed by 2-bromobiphenyl (0.50 g, 2.2 mmol), DMF (11.5 mL) and Pd(PPh_3_)_4_ (0.13 g, 0.1 mmol). The reaction mixture was stirred at 0 °C for 5 h. NaOH (1 M, 10 mL) was added and the product was extracted with DCM. The organic extract was washed with water and brine and dried with Na_2_SO_4_. The crude product was purified by flash chromatography with hexanes to yield *o*,*p*’-quaterphenyl as a white solid (0.55 g, mp 107–109 °C, lit 117–120 °C, 90% yield). ^1^H NMR (400 MHz, CDCl_3_) δ 7.61–7.57 (m, 2H), 7.50–7.40 (m, 8H), 7.35–7.29 (m, 1H), 7.26–7.17 (m, 7H).

**Suzuki–Miyaura coupling to form o,*m*’-Quaterphenyl (16)** [35]**:** 2-Bromobiphenyl (0.20 g, 0.80 mmol), biphenyl-3-boronic acid (0.25 g, 1.3 mmol), and Pd(PPh_3_)_4_ (0.02 g, 0.02 mmol) were dissolved in DMF (10 mL). The reaction mixture was cooled to 0 °C and purged with nitrogen. A solution of K_2_CO_3_ (0.20 g, 1.5 mmol) and water (5 mL) was added via syringe and the reaction mixture was stirred at 0 °C for 5 h. The product was extracted with DCM, and washed with water, NaOH, and HCl. The organic extract was then washed with water and brine and dried with Na_2_SO_4_. The crude product was purified by flash chromatography with hexanes to yield *o*,*m*’-quaterphenyl as a white solid (0.085 g, mp 84–86 °C, lit 90–91 °C, 33% yield). ^1^H NMR (400 MHz, CDCl_3_) δ 7.52–7.47 (m, 1H), 7.47–7.40 (m, 4H), 7.38–7.29 (m, 6H), 7.29–7.26 (m, 1H), 7.26–7.22 (m, 3H), 7.21–7.15 (m, 3H).

**Synthesis of *o*,*o*’-quaterphenyl (17)** [36]**:** A solution of 2-bromobiphenyl (0.15 g, 0.65 mmol), magnesium turnings (0.02 g, 0.69 mmol), and THF (2 mL) was stirred at ambient temperature overnight under a nitrogen atmosphere. Additional THF (4 mL) was added and the reaction mixture was cooled to −78 °C via a dry ice/acetone bath. TiCl_4_ (0.14 g, 0.65 mmol) was added dropwise via syringe and the reaction mixture was warmed to 0 °C via an ice/water bath and stirred for 1 h. The product was extracted with ethyl acetate (3x) and dried with Na_2_SO_4_. The crude product was purified by chromatography with hexanes to yield *o*,*o*’-quaterphenyl as a white solid (0.040 g, mp 110–113 °C, *lit* 116–118 °C, 20% yield). ^1^H NMR (400 MHz, CDCl_3_) δ 7.4–7.40 (m, 2H), 7.38–7.29 (m, 4H), 7.18–7.14 (m, 2H), 7.10–7.05 (m, 2H), 7.03–6.96 (m, 4H), 6.62–6.59 (m, 4H).

### Rearrangement of quaterphenyl isomers in a microwave reactor

***p*,*p*’-Quaterphenyl (12):** 150 °C, 30 min. Compound **12** (17 mg, 0.06 mmol) was heated in a MW reactor (150 °C, 30 min) according to the general rearrangement procedure. The crude product was purified via CombiFlash with hexanes to yield *m*,*p*’-quaterphenyl (**13**) as an off-white solid (12 mg, 70% yield).

***m*,*p*’-Quaterphenyl (13):** 150 °C, 30 min. Compound **13** (17 mg, 0.05 mmol) was heated in a MW reactor (150 °C, 30 minutes) according to the general rearrangement procedure. The crude product was filtered over a silica plug with hexanes to yield **13** as an off-white solid (15 mg, 88% yield).

150 °C, 1 h. **13** (16 mg, 0.05 mmol) was heated in a MW reactor (150 °C, 1 h) according to the general rearrangement procedure. The crude product was filtered over a silica plug with hexanes to yield **13** as an off-white solid (12 mg, 75% yield).

***m*,*m*’-Quaterphenyl (14):** 150 °C, 30 min. Compound **14** (16 mg, 0.05 mmol) was heated in a MW reactor (150 °C, 30 minutes) according to the general rearrangement procedure. The crude product was purified via CombiFlash with hexanes to yield an off-white solid (13 mg, 81% yield) consisting of **13** (50%) and **14** (50%).

150 °C, 1 h*.* Compound **12** (10 mg, 0.03 mmol) was heated in a MW reactor (150 °C, 1 h) according to the general rearrangement procedure. The crude product was filtered over a silica plug with hexanes to yield an off-white solid (9 mg, 90% yield) consisting of **13** (67%) and **14** (33%).

***o*,*p*’-Quaterphenyl:** Compound **15** (10 mg, 0.03 mmol) was heated in a MW reactor (150 °C, 30 minutes) according to the general rearrangement procedure. The crude product was purified via CombiFlash with hexanes to yield an off-white solid (7 mg, 70% yield) consisting of **13** (81%) and **14** (19%). Minor products were eluted with ethyl acetate. MALDI–TOF–MS analysis indicated oligomerization (*m*/z = 344, 673).

***o*,*m*’-Quaterphenyl:** Compound **16** (28 mg, 0.09 mmol) was heated in a MW reactor (150 °C, 30 minutes) according to the general rearrangement procedure. The crude product was filtered over a silica plug with hexanes to yield an off-white solid (18 mg, 64% yield) consisting of **13** (57%) and **14** (43%).

**o,o’-Quaterphenyl:** Compound **17** (6 mg, 0.02 mmol) was heated in a MW reactor (150 °C, 30 minutes) according to the general rearrangement procedure. The crude product was filtered over a silica plug with hexanes to yield an off-white solid (4 mg, 67% yield) consisting of **13** (60%) and **14** (40%).

**Computational methods:** DFT computations on carbocation intermediates and transition states were carried out with the B3LYP functional and 6-31+G(d,p) basis set, using the polarizable continuum model in dichloroethane to model solvation [42]. Each stationary point was characterized as a minimum or transition state by vibrational frequency analysis but the large number of reaction paths precluded calculation of intrinsic reaction coordinates. Reported relative energies are from free energy calculations at 298 K.

## Supporting Information

File 1 Selected NMR spectra, MALDI spectrum of the product mixture, Cartesian coordinates, and summary energetics for all stationary points.
