# Supplementary material for: Acid-catalyzed rearrangements in arenes: interconversions in the quaterphenyl series
Source: Beilstein J Org Chem. 2019 Nov 6;15:2655–63. doi: 10.3762/bjoc.15.258 (PMC6880835; doi:10.3762/bjoc.15.258)
Supplement: File 1 — Selected NMR spectra, MALDI spectrum of the product mixture, Cartesian coordinates, and summary energetics for all stationary points. [file Beilstein_J_Org_Chem-15-2655-s001.pdf]

BEILSTEIN JOURNAL OF ORGANIC CHEMISTRY

Supporting Information

for

Acid-catalyzed rearrangements in arenes: interconversions in the quaterphenyl series

Sarah L. Skraba-Joiner, Carter J. Holt and Richard P. Johnson

Beilstein J. Org. Chem. **2019,** *15,* 2655–2663. doi:10.3762/bjoc.15.258

Selected NMR spectra, MALDI spectrum of the product mixture, Cartesian coordinates, and summary energetics for all stationary points

License and Terms: This is a supporting information file under the terms of the Creative Commons Attribution License (http://creativecommons.org/licenses/by/4.0). Please note that the reuse, redistribution and reproduction in particular requires that the authors and source are credited.
The license is subject to the *Beilstein Journal of Organic Chemistry* terms and conditions: (https://www.beilstein-journals.org/bjoc)

Table of contents

I. Experimental details and selected NMR spectra SI-1

II. Complete Gaussian reference SI-7

III. Table of energies SI-7

IV. Heat of formation estimates for neutral quaterphenyl isomers SI-8

V. Cartesian coordinates for optimized structures SI-9

I. Experimental details and selected NMR spectra

Rearrangement of quaterphenyl isomers in a microwave reactor

Interconversion of quaterphenyl isomers was observed by acid-catalyzed rearrangements performed in a microwave reactor. Reaction products were purified by chromatography with hexanes to give the various isomers without oligomeric impurities. Analysis of ^{1}H NMRs for pure samples of *m*-, *p*-, and *m,p*-quaterphenyl are easily distinguished by the appearance of a triplet at 8.9 ppm found in both *m*- and *m*,*p*-quaterphenyl (**14** and **13**), but not in *p*-quaterphenyl (**12**). This same proton resonance is useful in differentiating *m*-quaterphenyl from *m*,*p*-quaterphenyl. In the former case it integrates in a 2:15 ratio relative to the other aromatic protons, whereas in the case of *m*-,*p*-quaterphenyl it integrates in a 1:17 ratio. Using this as a spectroscopic tag the clean conversion of *p*-quaterphenyl to *m*, *p*-quaterphenyl can be seen (**S4**). However, when *m*,*p*-quaterphenyl is subjected to the acid-catalyzed rearrangement conditions significant oligomerization is observed, but no formation of other isomers (**S5**).

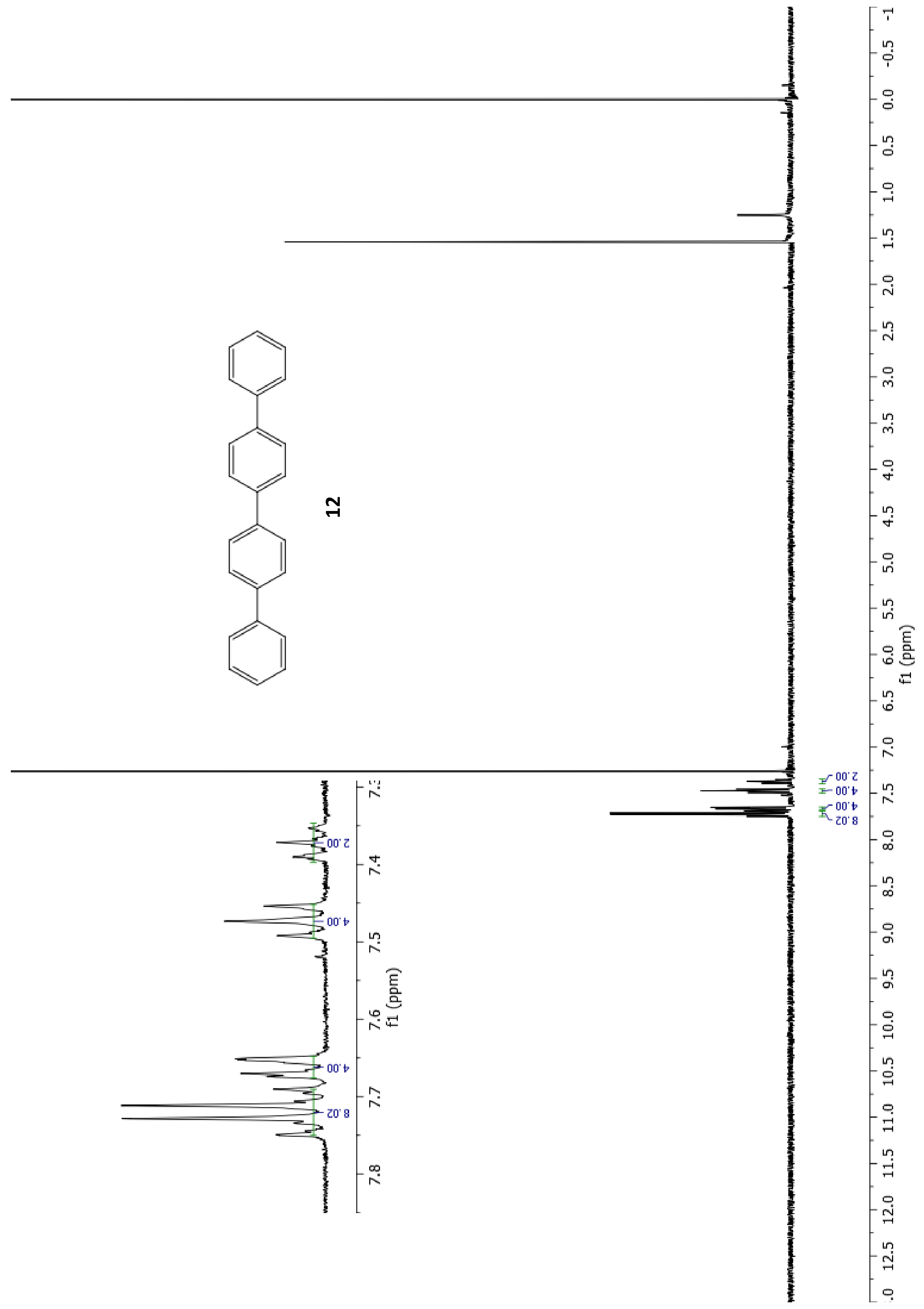
12
f1 (ppm)
2.00
4.00
4.00
8.02

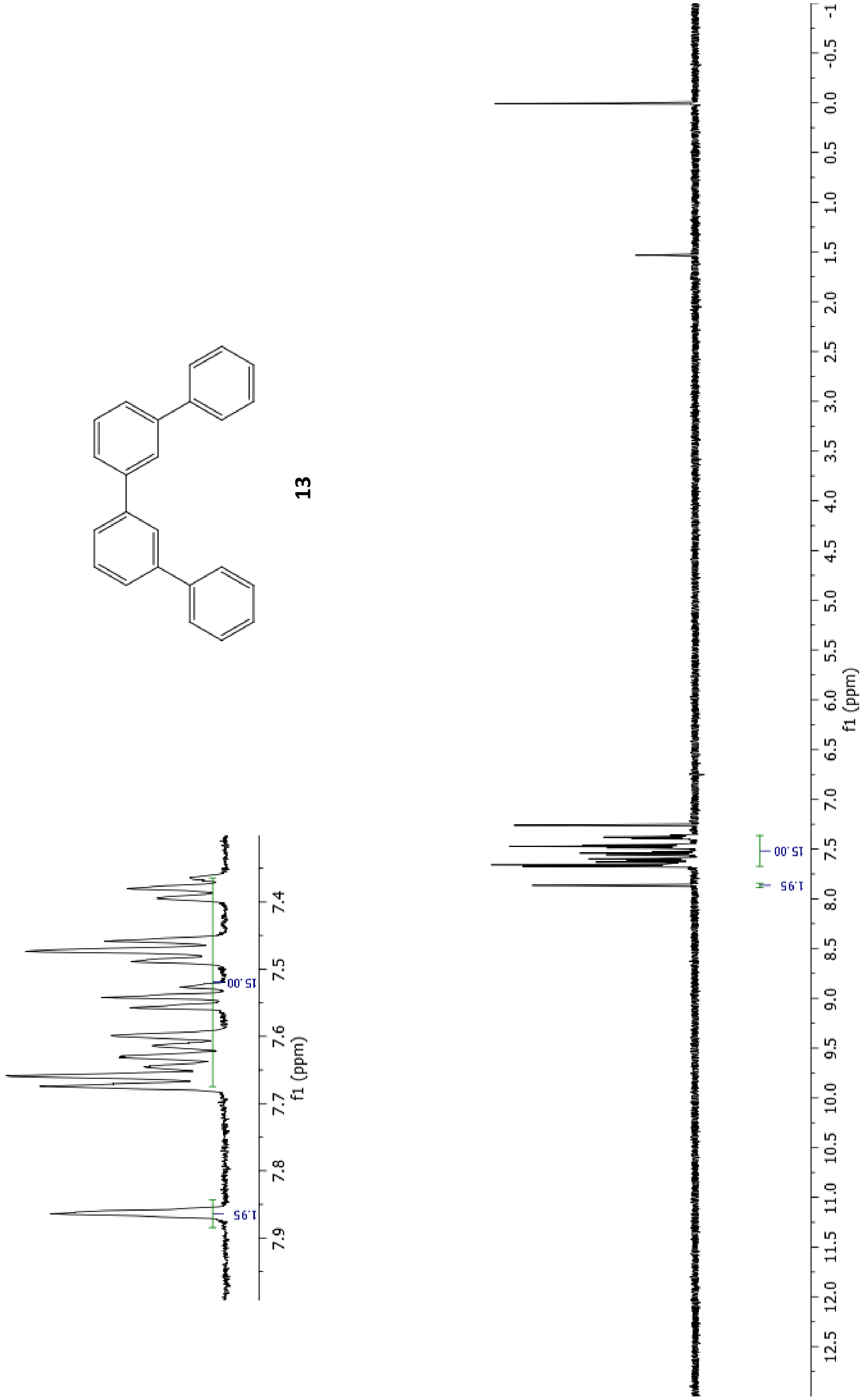
13
f1 (ppm)
15.00
1.95

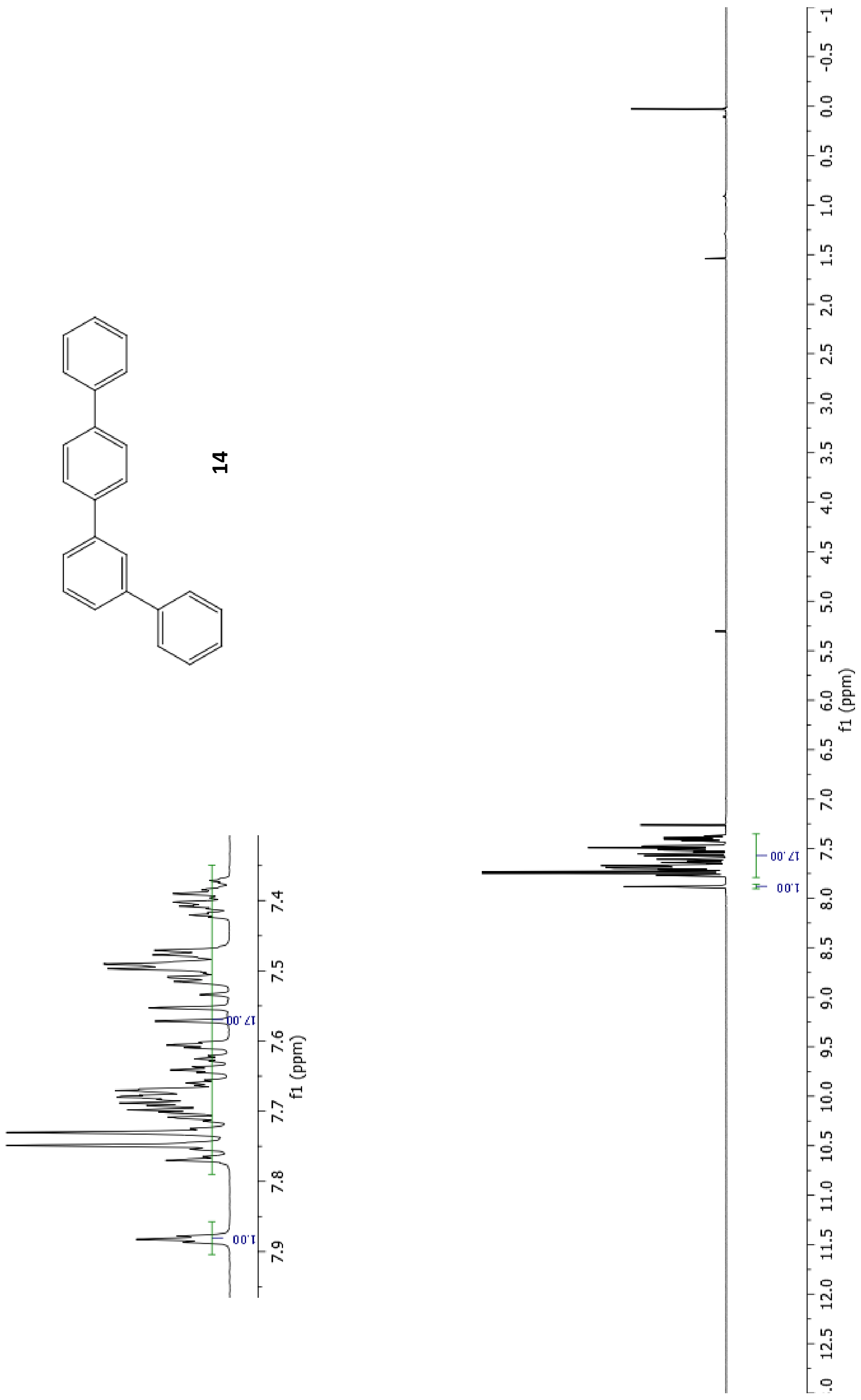
14
f1 (ppm)
17.00
1.00

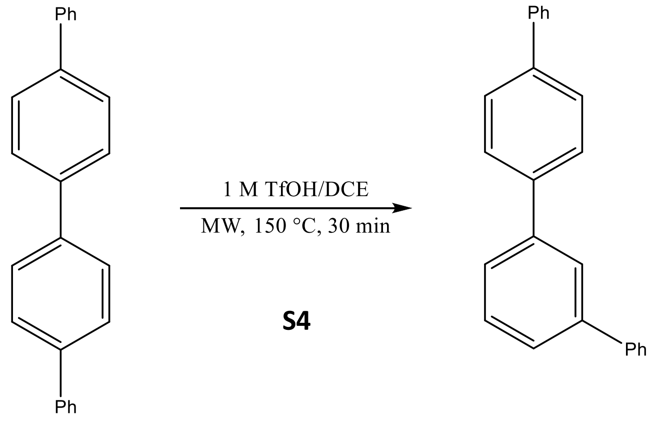
Ph
Ph
1 M TfOH/DCE
MW, 150 °C, 30 min
Ph
Ph

S4

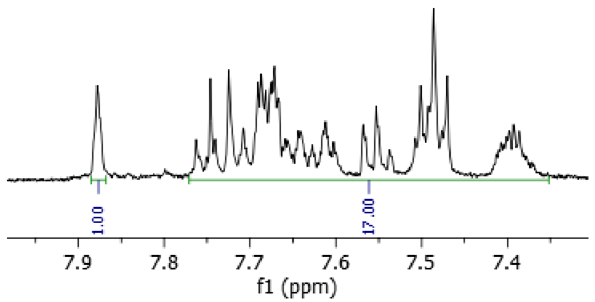
1.00
17.00
7.9
7.8
7.7
7.6
7.5
7.4
f1 (ppm)

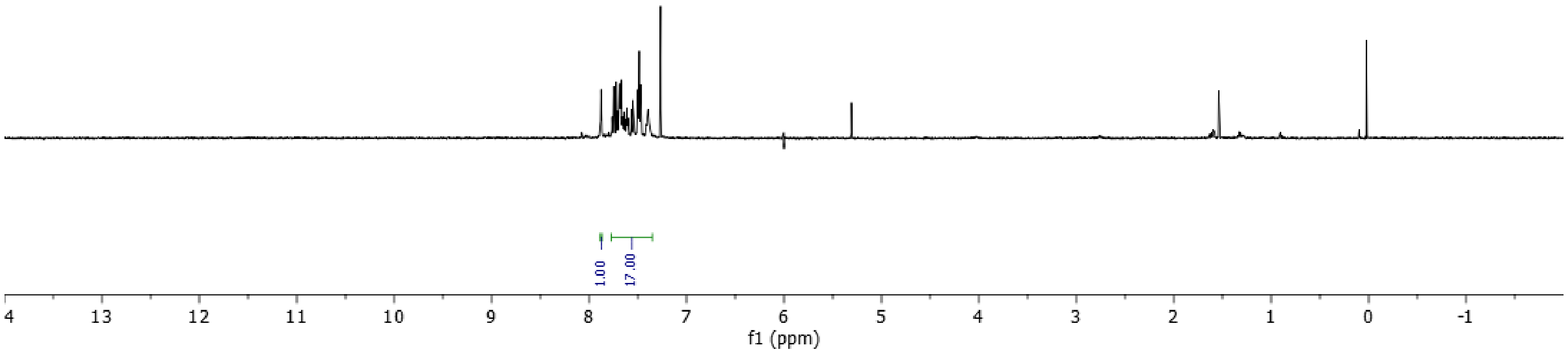
1.00
17.00
f1 (ppm)

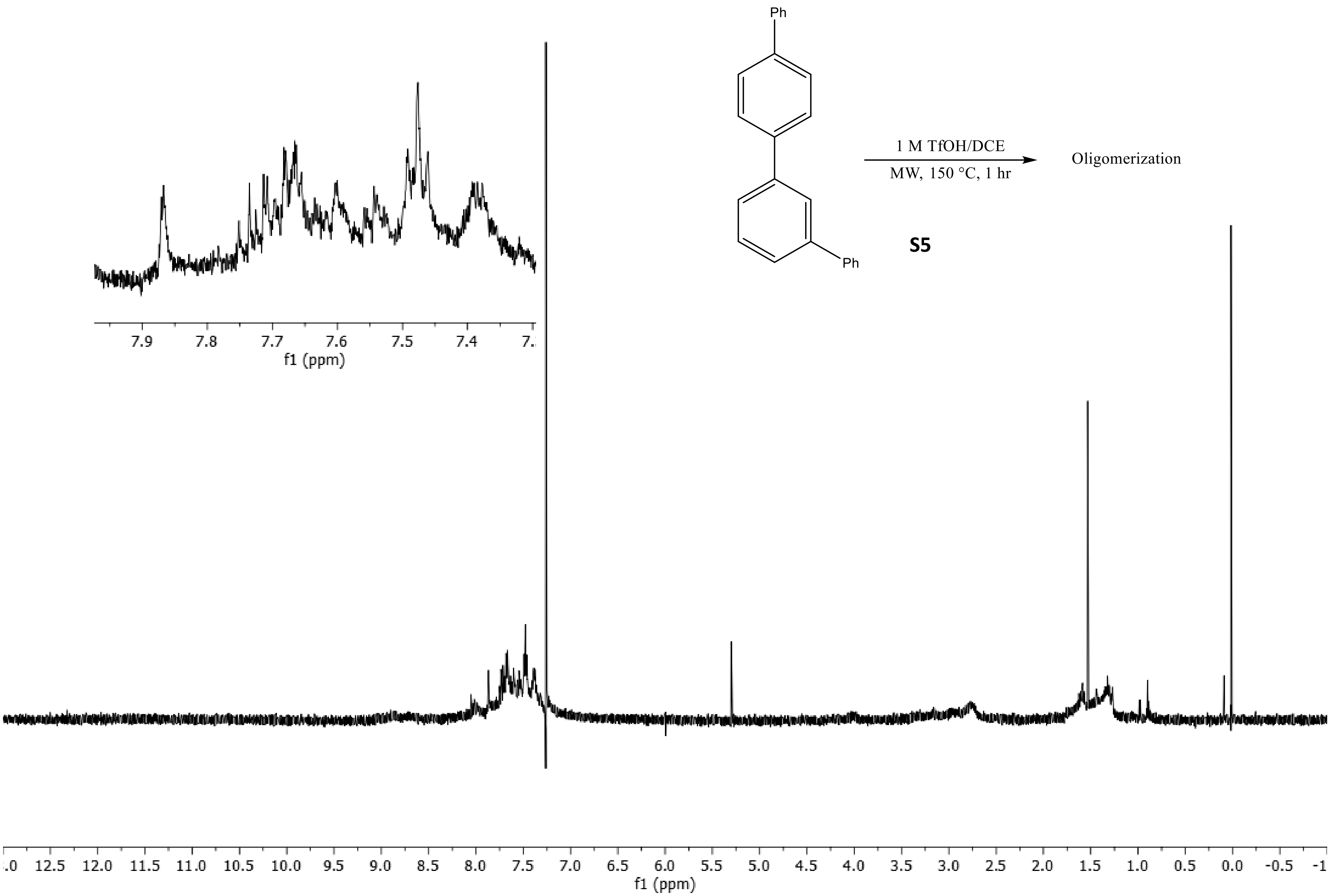
Ph
Ph
1 M TfOH/DCE
MW, 150 °C, 1 hr
Oligomerization
S5
7.9
7.8
7.7
7.6
7.5
7.4
7.
f1 (ppm)
.0
12.5
12.0
11.5
11.0
10.5
10.0
9.5
9.0
8.5
8.0
7.5
7.0
6.5
6.0
5.5
5.0
4.5
4.0
3.5
3.0
2.5
2.0
1.5
1.0
0.5
0.0
-0.5
-1
f1 (ppm)

II. Complete Gaussian reference:

Gaussian 09, Revision B.01, Frisch, M. J.; Trucks, G. W.; Schlegel, H. B.; Scuseria, G. E.; Robb, M. A.; Cheeseman, J. R.; Scalmani, G.; Barone, V.; Mennucci, B.; Petersson, G. A.; Nakatsuji, H.; Caricato, M.; Li, X.; Hratchian, H. P.; Izmaylov, A. F.; Bloino, J.; Zheng, G.; Sonnenberg, J. L.; Hada, M.; Ehara, M.; Toyota, K.; Fukuda, R.; Hasegawa, J.; Ishida, M.; Nakajima, T.; Honda, Y.; Kitao, O.; Nakai, H.; Vreven, T.; Montgomery, Jr., J. A.; Peralta, J. E.; Ogliaro, F.; Bearpark, M.; Heyd, J. J.; Brothers, E.; Kudin, K. N.; Staroverov, V. N.; Kobayashi, R.; Normand, J.; Raghavachari, K.; Rendell, A.; Burant, J. C.; Iyengar, S. S.; Tomasi, J.; Cossi, M.; Rega, N.; Millam, J. M.; Klene, M.; Knox, J. E.; Cross, J. B.; Bakken, V.; Adamo, C.; Jaramillo, J.; Gomperts, R.; Stratmann, R. E.; Yazyev, O.; Austin, A. J.; Cammi, R.; Pomelli, C.; Ochterski, J. W.; Martin, R. L.; Morokuma, K.; Zakrzewski, V. G.; Voth, G. A.; Salvador, P.; Dannenberg, J. J.; Dapprich, S.; Daniels, A. D.; Farkas, Ö.; Foresman, J. B.; Ortiz, J. V.; Cioslowski, J.; Fox, D. J. Gaussian, Inc., Wallingford CT, 2009.

III. Table of energies

Molecule	*E* (au)	ZPE (au)	*E*corr (au)	*E* (kcal/mol)	Free Energy (au)	Free Energy (kcal/mol)
**12**	-925.4874885	0.342997	-925.144491	-580537.4195	-925.191697	-580567.0418
**12a**	-925.8752005	0.353958	-925.521243	-580773.8352	-925.569853	-580804.3385
**12b**	-925.8708929	0.353906	-925.516987	-580771.1645	-925.566156	-580802.0186
**12c**	-925.8640952	0.354508	-925.509587	-580766.5209	-925.559152	-580797.6235
**12d**	-925.8612699	0.354457	-925.506813	-580764.7802	-925.555905	-580795.5859
**13**	-925.4870423	0.342747	-925.144295	-580537.2966	-925.192917	-580567.8073
**13a**	-925.8791421	0.354191	-925.524951	-580776.162	-925.574603	-580807.3191
**13b**	-925.8681353	0.353334	-925.514802	-580769.7934	-925.565053	-580801.3264
**13c**	-925.8606282	0.354132	-925.506496	-580764.5813	-925.556575	-580796.0064
**13d**	-925.8484096	0.35327	-925.495139	-580757.4547	-925.544727	-580788.5716
**13e**	-925.8610043	0.354215	-925.50679	-580764.7658	-925.557005	-580796.2762
**13f**	-925.8480189	0.353404	-925.494615	-580757.1259	-925.544208	-580788.246
**14**	-925.4866369	0.342872	-925.143764	-580536.9633	-925.191712	-580567.0512
**14a**	-925.8770303	0.354233	-925.522797	-580774.8103	-925.571751	-580805.5295
**14b**	-925.8477739	0.353231	-925.494543	-580757.0807	-925.543984	-580788.1054
**14c**	-925.8478646	0.35295	-925.494915	-580757.3141	-925.545965	-580789.3485
**15**	-925.4807047	0.342543	-925.138162	-580533.448	-925.186652	-580563.876
**15a**	-925.8680674	0.35375	-925.514317	-580769.4891	-925.562627	-580799.8041
**15b**	-925.8638066	0.353385	-925.510422	-580767.0449	-925.559574	-580797.8883
**15c**	-925.863973	0.353483	-925.51049	-580767.0876	-925.559086	-580797.5821
**15d**	-925.8586383	0.354134	-925.504505	-580763.3319	-925.554367	-580794.6208
**15e**	-925.8542218	0.353876	-925.500346	-580760.7221	-925.549569	-580791.61
**15f**	-925.8573647	0.353979	-925.503386	-580762.6297	-925.552715	-580793.5842

**15g**	-925.8562531	0.354245	-925.502008	-580761.765	-925.551463	-580792.7985
**16**	-925.4803733	0.342477	-925.137896	-580533.2811	-925.186257	-580563.6281
**16a**	-925.8708235	0.35373	-925.517094	-580771.2317	-925.565861	-580801.8334
**16b**	-925.8617296	0.353245	-925.508485	-580765.8294	-925.557372	-580796.5065
**16c**	-925.8557336	0.353915	-925.501818	-580761.6458	-925.551419	-580792.7709
**16d**	-925.8428459	0.352931	-925.489915	-580754.1766	-925.53966	-580785.392
**16e**	-925.8560426	0.354195	-925.501848	-580761.6646	-925.550599	-580792.2564
**16f**	-925.8451501	0.352793	-925.492357	-580755.7089	-925.541801	-580786.7355
**17**	-925.474039	0.342329	-925.13171	-580529.3993	-925.178954	-580559.0454
**17a**	-925.8587339	0.353405	-925.505329	-580763.849	-925.553035	-580793.785
**17b**	-925.8563084	0.353205	-925.503103	-580762.4522	-925.551085	-580792.5613
**17c**	-925.8477796	0.353984	-925.493796	-580756.6119	-925.542314	-580787.0575
**17d**	-925.8509692	0.353958	-925.497011	-580758.6294	-925.545603	-580789.1213
**TS7**	-925.8329804	0.353436	-925.479545	-580747.6693	-925.527421	-580777.712
**TS8**	-925.834207	0.353555	-925.480652	-580748.3639	-925.528225	-580778.2165
**TS9**	-925.8322389	0.353423	-925.478816	-580747.2118	-925.52689	-580777.3787
**TS10**	-925.825763	0.353242	-925.472521	-580743.2617	-925.519923	-580773.0069
**TS11**	-925.8333134	0.353341	-925.479972	-580747.9372	-925.527875	-580777.9968
**TS12**	-925.8250287	0.353578	-925.47145	-580742.5896	-925.517955	-580771.7719
**TS13**	-925.8345467	0.353437	-925.48111	-580748.6513	-925.529016	-580778.7128
**TS14**	-925.8362703	0.353736	-925.482534	-580749.5449	-925.529975	-580779.3146
**TS15**	-925.8318874	0.353309	-925.478578	-580747.0625	-925.526457	-580777.107
**TS16**	-925.8267528	0.352959	-925.473794	-580744.0605	-925.521973	-580774.2933
**TS17**	-925.8184324	0.353404	-925.465028	-580738.5597	-925.512234	-580768.182
**TS18**	-925.8281896	0.353604	-925.474586	-580744.5575	-925.521429	-580773.9519

IV. Heat of formation estimates for neutral quaterphenyls

T1 Method for predicting heats of formation: Ohlinger, W. S.; Klunzinger, P. E.; Deppmeier, B. J.; Hehre, W. J. *The Journal of Physical Chemistry A* **2009**, *113* (10), 2165-2175.

Isomer	Number	T1 HF kJ/mol	*E*rel kJ/mol	E_{rel} (kcal/mol)	Etot(au) M062X/6-311+G(d,p)	E_{rel} (kcal/mol)
p,p'	**12**	337.5	5.74	1.37	-925.249855	1.07
m,p'	**13**	337.43	5.67	1.35	-925.24974	1.14
m,m'	**14**	337.14	5.37	1.28	-925.249485	1.3
o,m'	**16**	333.96	2.19	0.52	-925.250248	0.82
o,p'	**15**	339.04	7.27	1.74	-925.247674	2.43
o,o'	**17**	331.77	0	0	-925.251553	0

V. Cartesian coordinates for optimized structures

12

Charge = 0 Multiplicity = 1
C,3.5959143028,0.,0.
C,0.742443976,0.,0.
C,2.8662156069,1.1436468936,-0.3720228515
C,2.8662156069,-1.1436468936,0.3720228515
C,1.4724497898,-1.1437565544,0.3720106038
C,1.4724497898,1.1437565544,-0.3720106038
H,3.3930733063,2.036161628,-0.6960706231
H,3.3930733063,-2.036161628,0.6960706231
H,0.9461176636,-2.0363815729,0.696551614
H,0.9461176636,2.0363815729,-0.696551614
C,5.0822969265,0.,0.
C,7.908285084,0.,0.
C,5.8062148487,-1.1393896923,-0.3986893173
C,5.8062148487,1.1393896923,0.3986893173
C,7.2027826861,1.1400235049,0.3980504462
C,7.2027826861,-1.1400235049,-0.3980504462
H,5.2730685385,-2.0244325509,-0.7329905262
H,5.2730685385,2.0244325509,0.7329905262
H,7.7393205092,2.0289430787,0.7174605948
H,7.7393205092,-2.0289430787,-0.7174605948
H,8.9943083903,0.,0.
C,-0.742443976,0.,0.
C,-3.5959143028,0.,0.
C,-1.4724497898,1.1437565544,0.3720106038
C,-1.4724497898,-1.1437565544,-0.3720106038
C,-2.8662156069,-1.1436468936,-0.3720228515
C,-2.8662156069,1.1436468936,0.3720228515
H,-0.9461176636,2.0363815729,0.696551614
H,-0.9461176636,-2.0363815729,-0.696551614
H,-3.3930733063,-2.036161628,-0.6960706231
H,-3.3930733063,2.036161628,0.6960706231
C,-5.0822969265,0.,0.
C,-7.908285084,0.,0.
C,-5.8062148487,1.1393896923,-0.3986893173
C,-5.8062148487,-1.1393896923,0.3986893173
C,-7.2027826861,-1.1400235049,0.3980504462
C,-7.2027826861,1.1400235049,-0.3980504462
H,-5.2730685385,2.0244325509,-0.7329905262
H,-5.2730685385,-2.0244325509,0.7329905262
H,-7.7393205092,-2.0289430787,0.7174605948
H,-7.7393205092,2.0289430787,-0.7174605948
H,-8.9943083903,0.,0.

12a

Charge = 1 Multiplicity = 1

C,0.000000012,0.,-3.40713886
C,0.0000000023,0.,-0.552540631
C,-1.1936194689,-0.212968615,-2.6683853078
C,1.1936194879,0.212968615,-2.6683852997
C,1.1882068513,0.2248098906,-1.2854347524
C,-1.1882068417,-0.2248098906,-1.2854347604
H,-2.1247416997,-0.4242088381,-3.1803064225
H,2.1247417221,0.4242088381,-3.1803064081
H,2.1122152598,0.4416729211,-0.761648823
H,-2.1122152537,-0.4416729211,-0.7616488373
C,-0.0000000026,0.,0.9217880855
C,-0.0000000123,0.,3.7713209162
C,-1.1437544623,0.3829204138,1.6513616142
C,1.1437544521,-0.3829204138,1.6513616219
C,1.1404977021,-0.3879697269,3.0421196384
C,-1.1404977217,0.3879697269,3.0421196307
H,-2.0336462084,0.7198778067,1.1299343322
H,2.0336462017,-0.7198778067,1.129934346
H,2.0269906163,-0.7260652809,3.5690010378
H,-2.0269906394,0.7260652809,3.5690010241
C,-0.0000000173,0.,5.2555707303
C,-0.0000000268,0.,8.0783163809
C,-1.1516149466,-0.3656886028,5.9778422143
C,1.1516149072,0.3656886028,5.9778422221
C,1.1510597592,0.3669454001,7.3738620678
C,-1.1510598081,-0.3669454001,7.37386206
H,-2.0458606501,-0.6750656861,5.445382663
H,2.0458606142,0.6750656861,5.4453826768
H,2.0477849112,0.662141798,7.9108557208
H,-2.0477849637,-0.662141798,7.910855707
H,-0.0000000305,0.,9.1642227306
C,0.0000000169,0.,-4.8540734624
C,0.0000000266,0.,-7.744441594
C,1.216285376,-0.2101591966,-5.5901291686
C,-1.2162853373,0.2101591966,-5.5901291768
C,-1.2256765076,0.2177960587,-6.9501586967
C,1.2256765555,-0.2177960587,-6.9501586884
H,2.1369049874,-0.4034870156,-5.0547807888
H,-2.1369049523,0.4034870156,-5.0547808032
H,-2.1527317446,0.3992826264,-7.4855420302
H,2.1527317961,-0.3992826264,-7.4855420156
H,-0.1506548634,-0.8450122461,-8.4404853267
H,0.1506549213,0.8450122461,-8.4404853257

12b

Charge = 1 Multiplicity = 1
C,0.0039969851,-0.0376356696,-3.5942703435
C,-0.0087952124,0.0315660984,-0.6636011079
C,0.1185965688,-1.2050097732,-2.8919641295
C,-0.1131994835,1.162447145,-2.8185273109

C,-0.1154151544,1.2010165978,-1.4320487918
C,0.1122841978,-1.2561597604,-1.4126058693
H,0.2318458407,-2.1548174704,-3.4055773613
H,-0.2252696619,2.1046551181,-3.3471406656
H,-0.2263412412,2.1677757939,-0.9588043894
C,-0.0145633387,0.0402345241,0.7849268366
C,-0.009083471,0.0465077021,3.6414783182
C,-0.0161672413,-1.1679768581,1.527854397
C,-0.009369137,1.2524607486,1.5229920204
C,-0.005245361,1.252284034,2.9072411469
C,-0.0152015341,-1.1634594292,2.9116911082
H,-0.0382330222,-2.1257716044,1.0217605485
H,0.0177830243,2.2086063509,1.0155723546
H,0.0311945737,2.2015631257,3.429974953
H,-0.0478789996,-2.1105362096,3.4386424316
C,-0.0062597014,0.0490234633,5.1193746728
C,0.0020672898,0.0556299237,7.9385328874
C,0.6384222548,-0.9751847458,5.8416166877
C,-0.6470817773,1.0763740668,5.840574819
C,-0.6461377607,1.0762719444,7.2351235422
C,0.6458898403,-0.9684463737,7.2361580628
H,1.1618977637,-1.7647064563,5.3120767831
H,-1.1736879014,1.863285182,5.3102777794
H,-1.1565045636,1.8696618853,7.772604415
H,1.159446782,-1.7593037006,7.7743362275
H,0.0053456947,0.0582534918,9.0242639555
C,-0.001781692,0.0138640498,-5.0789942911
C,0.0074401581,0.0975217905,-7.8910496283
C,0.6668936886,1.0448320458,-5.7616225431
C,-0.6704629311,-0.971516935,-5.8257773869
C,-0.664068081,-0.9299257442,-7.2210477932
C,0.6725359738,1.0843170797,-7.1572236201
H,1.2065702173,1.8061172105,-5.205715207
H,-1.2171036126,-1.7593048478,-5.3158198226
H,-1.1918210221,-1.6941466091,-7.7834525344
H,1.2011841027,1.8823205242,-7.6695934339
H,0.0100282087,0.1302645382,-8.9762698226
H,1.0209049213,-1.7888417175,-1.087380643
H,-0.6946558749,-1.9441103999,-1.1085873621

12c

Charge = 1 Multiplicity = 1
C,-1.0383478134,-0.1523715592,-3.6320337598
C,-0.6339455843,-0.0903271926,-0.7453222901
C,-1.0929496779,1.096188831,-2.8261747417
C,-0.7296479233,-1.3625851713,-2.8243105275
C,-0.5579939007,-1.3260938516,-1.4769598834
C,-0.9065060495,1.1151938483,-1.4804023842
H,-1.3131051722,2.0185177791,-3.3555125633
H,-0.6440073897,-2.305673239,-3.3559158307

H,-0.3163822571,-2.2431819657,-0.955590185
H,-0.9952084936,2.0559042079,-0.9528444931
C,-0.1462448625,-0.0203580989,-4.8795731154
C,1.492281571,0.217003841,-7.1427644783
C,-0.6955308956,-0.1325462871,-6.1618135164
C,1.2289848882,0.2121359253,-4.734092339
C,2.043843687,0.3298612528,-5.8622357651
C,0.1222251782,-0.0141245217,-7.2902031886
H,-1.7602518264,-0.3115934035,-6.2837810798
H,1.6658061434,0.3018117511,-3.7430780839
H,3.1076906581,0.5096159699,-5.7400683926
H,-0.3137516688,-0.1023939591,-8.2806842887
H,2.1267977854,0.3090542239,-8.0189450082
C,-0.4416657946,-0.0620913979,0.6870904923
C,-0.0598765712,-0.008010339,3.5150040395
C,-0.1534801275,1.149137151,1.3717317552
C,-0.5330290151,-1.2450188492,1.4689558839
C,-0.3577487333,-1.2137773425,2.8402059216
C,0.0416608172,1.1702476766,2.7405084048
H,-0.0316129255,2.0748403545,0.8228904994
H,-0.7931771746,-2.18966487,1.0074172531
H,-0.4833054499,-2.1304517387,3.4052514689
H,0.3067618719,2.1073126022,3.2169691238
C,0.137573964,0.0194327481,4.978001997
C,0.5137095688,0.071586413,7.7706129193
C,-0.218667165,1.1553849625,5.7330074508
C,0.6866040528,-1.0898170399,5.6529331296
C,0.8772942831,-1.0605636595,7.0337517173
C,-0.037369872,1.1777924244,7.1152356545
H,-0.6675953283,2.0132412241,5.242940614
H,0.99465411,-1.9672567028,5.0936390669
H,1.3148443374,-1.9191205174,7.5338518864
H,-0.331286345,2.056312549,7.6813399592
H,0.658605341,0.0915674208,8.8464232906
H,-2.066650191,-0.3051214401,-4.0154565733

12d

Charge = 1 Multiplicity = 1
C,-0.3376206934,-0.1849538344,-3.3918628213
C,-1.3502145783,-0.890411962,-0.7616897276
C,0.084922261,-1.393540182,-2.7443919199
C,-1.2676769084,0.6696999174,-2.7115289193
C,-1.7448765485,0.3487455962,-1.4785771406
C,-0.3709867681,-1.7239093874,-1.5057138896
H,0.8154999233,-2.0306036941,-3.2264006512
H,-1.6228953219,1.5687755498,-3.1984594158
H,-2.462711714,0.9986935382,-0.9872679579
H,-0.0192050188,-2.6294724772,-1.0202889416
C,-0.9352711015,-0.6312524561,0.697037893
C,-0.1529730488,-0.1416387205,3.3729376934

C,-1.658887079,-1.1823523619,1.7593169516
C,0.1826074802,0.1662874305,0.9762417514
C,0.5670509662,0.4049162713,2.2944777268
C,-1.2727344868,-0.9387614164,3.0783859085
H,-2.5333673396,-1.7968684215,1.5641623455
H,0.7628680936,0.5971301477,0.1649987719
H,1.4500516524,1.0066546146,2.4852591593
H,-1.8650097126,-1.357217457,3.8859668897
C,0.258563516,0.1165013925,4.7783126668
C,1.0423328201,0.6057938803,7.4456118879
C,0.166098854,-0.8926372602,5.7542939858
C,0.7517733933,1.3758831287,5.1661742382
C,1.1393990329,1.6180354405,6.4857473587
C,0.5538071603,-0.6506690991,7.0738758697
H,-0.1895203975,-1.8802530547,5.4758514776
H,0.8115751641,2.1785269671,4.4370880146
H,1.5093978702,2.6003710582,6.7648194158
H,0.4819599521,-1.446905172,7.8091050484
H,1.3439699629,0.7940494672,8.4716866994
C,0.1665951685,0.1664131065,-4.7089342895
C,1.1457237494,0.8487420397,-7.2571490817
C,0.1635213068,1.5137485208,-5.1516034977
C,0.6749644065,-0.828768939,-5.5820805012
C,1.1453234639,-0.4896252318,-6.844487018
C,0.6599023036,1.8484226664,-6.4048947448
H,-0.1866467956,2.3047245862,-4.4992268407
H,0.6618280292,-1.8722669055,-5.2907756609
H,1.5099117313,-1.2650769683,-7.509721295
H,0.6710445722,2.8863800309,-6.7199759252
H,1.5227769323,1.1119362307,-8.2404169684
H,-2.2728723638,-1.5032462203,-0.7059044559

13

Charge = 0 Multiplicity = 1
C,-0.4334403067,-0.8952029719,-0.1152543453
C,-3.1731871484,-0.1081837078,-0.006796841
C,-0.8768186341,0.2884392335,-0.7319556034
C,-1.3906542988,-1.6766228439,0.5566528925
C,-2.7292681019,-1.2916781963,0.6103274153
C,-2.2158379812,0.6724075067,-0.6798528349
H,-0.1733290723,0.9023323195,-1.2864200447
H,-1.0808149044,-2.5812793117,1.0712850489
H,-3.4319902935,-1.9052307125,1.1660711846
H,-2.5253416267,1.5764984333,-1.1956220631
C,0.9941654397,-1.3069158243,-0.1690856056
C,3.7023719508,-2.0766305812,-0.2402790331
C,1.3506514532,-2.6607787125,-0.3047357023
C,2.0207241972,-0.3524835154,-0.0709031335
C,3.3775150202,-0.7151352707,-0.1032082685
C,2.6942149859,-3.03691933,-0.3394888717

H,0.5774462507,-3.4163655824,-0.404713146
H,1.7578652024,0.689960249,0.0805343853
H,2.9575115571,-4.0836921183,-0.4614237253
H,4.7429567252,-2.3821295689,-0.2910250613
C,4.4433939276,0.3163099721,0.0190970122
C,6.4692227229,2.2717086052,0.2521292029
C,5.6103371334,0.063512711,0.7637359102
C,4.3133910062,1.5702785894,-0.6057784407
C,5.314529609,2.5374377572,-0.4906516618
C,6.612406781,1.0296709999,0.8786743159
H,5.7255232455,-0.8879450913,1.2744969551
H,3.4325299462,1.7825808325,-1.2044360501
H,5.1952015805,3.4956507773,-0.9883189702
H,7.5008571765,0.8144151854,1.4655445284
H,7.2478545012,3.0234840322,0.3418970708
C,-4.600682221,0.3026676749,0.0497683189
C,-7.3143801826,1.0844811008,0.1525582142
C,-4.9593812001,1.658300297,0.1713526282
C,-5.6323029318,-0.6524951825,-0.0181992694
C,-6.9734981322,-0.2665965327,0.0334661563
C,-6.3003790065,2.0451649706,0.2210473144
H,-4.1832927393,2.4139692518,0.2490975329
H,-5.3845335611,-1.7033242731,-0.1352453913
H,-7.752073153,-1.0216118508,-0.0287018599
H,-6.5521648883,3.097009436,0.3225909277
H,-8.3573000587,1.3849412431,0.1917689184

13a

Charge = 1 Multiplicity = 1
H,0.2103229344,1.7743697465,-4.9845306577
C,0.1552496689,1.6136656171,-3.9152223564
C,0.0893474849,1.0983056508,-1.0819838937
C,0.0765768396,0.2720602907,-3.3998990825
C,0.2014232569,2.6669326921,-3.0656668972
C,0.1692709351,2.5019978701,-1.5942944368
C,0.0435439363,0.0610012639,-2.0041806786
H,0.2733038065,3.6805550803,-3.448061182
H,-0.0801912617,-0.9539190685,-1.6519585497
C,0.0541117905,0.8558118841,0.356767262
C,-0.0155184376,0.385608934,3.1716434659
C,0.3157073284,-0.426618058,0.8971693544
C,-0.2278405825,1.8977268069,1.2713020001
C,-0.2670877643,1.666546782,2.6384268391
C,0.2831748906,-0.6522923947,2.2646339648
H,0.5883480152,-1.250859274,0.248883966
H,-0.4531404181,2.8965686748,0.9164549652
H,-0.5273127641,2.4847440379,3.3011916375
H,0.5235919071,-1.6417114246,2.6386489141
C,-0.0637698571,0.1385697844,4.6306622022
C,-0.1627029843,-0.3336102625,7.4089783159

C,-0.5274113782,-1.0909773542,5.1373285695
C,0.3492335486,1.1267726362,5.5452604724
C,0.3034109407,0.8909487612,6.9196833283
C,-0.5793552227,-1.3227382217,6.5121900858
H,-0.8750421217,-1.8598277828,4.4544000871
H,0.7336602345,2.0742985363,5.1807661214
H,0.6373630606,1.66139463,7.608154496
H,-0.9516230254,-2.273081993,6.8828739117
H,-0.2010080305,-0.5151825973,8.4788008339
C,0.0220680617,-0.864201385,-4.3213587151
C,-0.0902701702,-3.0463767593,-6.0950782885
C,-0.5224160187,-0.7149804572,-5.6178160125
C,0.5132854671,-2.1338863267,-3.9381129757
C,0.4661407941,-3.2081042208,-4.8206651943
C,-0.5886008006,-1.7990982776,-6.4879132467
H,-0.9316843385,0.2384916892,-5.9322070896
H,0.9711311582,-2.2703253728,-2.9647284756
H,0.8664970402,-4.1704339876,-4.5188171856
H,-1.0291925975,-1.673097083,-7.471521164
H,-0.1330725048,-3.8880576824,-6.7792983402
H,1.0515073277,3.0027472946,-1.1646864589
H,-0.6710300898,3.1002613903,-1.2062196021

13b

Charge = 1 Multiplicity = 1
H,0.5425924883,1.8232173504,-5.018700627
C,0.4647457135,1.6226196712,-3.9548481012
C,0.3196698921,1.1230261298,-1.2005746684
C,0.1804392414,0.3188899133,-3.5100152636
C,0.6706447779,2.6583892521,-3.0429018243
C,0.5967918156,2.4174828414,-1.6704810087
C,0.1135705745,0.0886060871,-2.1263078732
H,0.9029262482,3.6560035935,-3.4026415923
H,0.7786058495,3.2296673602,-0.9728612173
H,-0.150466608,-0.9017553122,-1.7676674258
C,0.2350762069,0.8521517432,0.2580232203
C,-0.0135931073,0.4721517337,3.1512318055
C,0.7211846693,-0.2907823988,0.8341990764
C,-0.3783832332,1.7951386952,1.1424135574
C,-0.4940847885,1.6229181959,2.5171412666
C,0.6243651156,-0.559237672,2.2843117254
H,1.2117865319,-1.05150648,0.2350639098
H,-0.801455352,2.6988720572,0.7134111444
H,-1.0017450746,2.3952723615,3.0801221388
C,-0.1224657536,0.2647963032,4.5885646923
C,-0.3269377534,-0.1287345515,7.3730270947
C,0.0907839705,-1.0130042544,5.1613691741
C,-0.4335408673,1.3411357389,5.4566944039
C,-0.5304219835,1.1445826727,6.829371067
C,-0.0156043877,-1.2056059234,6.5335173741

H,0.3201759981,-1.8680587904,4.5361741991
H,-0.5709707568,2.3419807186,5.0658316653
H,-0.7595856122,1.9842368454,7.4770788478
H,0.1384218174,-2.1951290857,6.9508911683
H,-0.4085158816,-0.2817035711,8.44454112
C,-0.0612747733,-0.7850609686,-4.4776677346
C,-0.525958943,-2.8772134574,-6.3143302224
C,-0.791697137,-0.5592191137,-5.6586033521
C,0.4322501055,-2.0800175339,-4.2360884324
C,0.2027260387,-3.1154049916,-5.1448749216
C,-1.0217559692,-1.5943891396,-6.56734738
H,-1.2010996721,0.4267064881,-5.8576630443
H,1.0203568411,-2.273701307,-3.343879306
H,0.6006833938,-4.1056495578,-4.9428007132
H,-1.5953041813,-1.3996452373,-7.4689151797
H,-0.7047997009,-3.6820644889,-7.0210836244
H,1.6357125593,-0.7838089034,2.6616946738
H,0.1061519574,-1.5261531641,2.4055801779

13c

Charge = 1 Multiplicity = 1
C,-0.5282323991,-1.136753689,0.3605333924
C,-3.3190460064,-0.52789312,0.892909271
C,-0.8925309376,0.0085766779,1.1442501688
C,-1.5718906279,-1.979696599,-0.1475513101
C,-2.8819227431,-1.6947757144,0.0844320834
C,-2.1967199549,0.3053220563,1.3929102167
H,-0.1184017953,0.6348532914,1.5685015968
H,-1.3182203063,-2.8303175828,-0.7667762451
H,-3.6582359961,-2.3302511529,-0.3311004883
H,-2.4518287062,1.1705902548,1.9974943208
C,0.8680692306,-1.4357402316,0.0844774718
C,3.5628916215,-1.9917502403,-0.4608388632
C,1.2731141229,-2.7449280215,-0.2760798415
C,1.8506496778,-0.4191401218,0.1601671467
C,3.1962301072,-0.6747024736,-0.1177021979
C,2.6112247895,-3.0126439916,-0.5338256601
H,0.5569568461,-3.5567421297,-0.3121209419
H,1.5540096472,0.5994170201,0.3785607411
H,2.9216925098,-4.0219652925,-0.7822567286
H,4.6056823069,-2.2225572871,-0.6543987826
C,4.2100109426,0.411010023,-0.0547381104
C,6.130402516,2.4699981855,0.0624859796
C,5.2422624496,0.4853669988,-1.0068503998
C,4.1581074431,1.385239618,0.9581962604
C,5.1099688531,2.4050407084,1.0162208225
C,6.1929058849,1.5065283238,-0.9489712504
H,5.2898940867,-0.2441327253,-1.8099562501
H,3.386390988,1.3322994234,1.7205430539
H,5.0592735294,3.1425574398,1.8117039692

H,6.9776513355,1.55245321,-1.6981545727
H,6.8708304354,3.2629101028,0.107910388
C,-4.420924739,0.2981555141,0.2032624519
C,-6.416014209,1.7971637549,-1.0719845376
C,-4.1229397448,1.0230513341,-0.9594910053
C,-5.7195084339,0.3266301397,0.7234678279
C,-6.714369935,1.0750094058,0.0863476159
C,-5.1184634466,1.7695199471,-1.5935785126
H,-3.1172938857,1.0072592596,-1.3711964567
H,-5.9582814573,-0.2330483857,1.6235336559
H,-7.719173586,1.0916263979,0.4972975785
H,-4.8795422041,2.3286441618,-2.4931471643
H,-7.1887893968,2.3783328128,-1.5657439024
H,-3.7917985727,-0.9572411332,1.7993513682

13d

Charge = 1 Multiplicity = 1
C,-0.4827613286,-0.819277156,0.1752117891
C,-3.2193981435,-0.0757889688,0.0455658738
C,-1.0262025022,0.0885757754,1.0994334564
C,-1.3256435541,-1.3489180346,-0.816232954
C,-2.66706903,-0.9799620391,-0.8803041848
C,-2.3696273923,0.4497639815,1.0362073211
H,-0.4081063415,0.4892097517,1.8974893967
H,-0.9305854754,-2.0267667662,-1.5674399584
H,-3.2853189884,-1.3798375271,-1.6775346748
H,-2.7688209709,1.1252540788,1.785951171
C,0.9470394164,-1.208239598,0.2486792067
C,3.6854052795,-2.0824224857,0.3532294251
C,1.3581513582,-2.5408865772,-0.0253595191
C,1.9404519566,-0.3018232564,0.5681316066
C,3.3607108723,-0.6843929504,0.6985819935
C,2.6967724734,-2.9743660402,0.0212978813
H,0.5944132971,-3.2733895743,-0.27090794
H,1.6960317534,0.7381469767,0.7621162116
H,2.9307037265,-4.0086390436,-0.2039808329
H,4.7252833523,-2.3882830194,0.4087989212
C,4.3622318979,0.3537117497,0.1745790743
C,6.1961622762,2.2186898977,-0.8222792014
C,4.3477014055,0.6954255578,-1.1852595244
C,5.2953783959,0.9461028707,1.0320798449
C,6.2111650731,1.8770828889,0.5324586882
C,5.2631708284,1.6268502912,-1.6797333311
H,3.6266455469,0.2373509212,-1.8569185711
H,5.3115090742,0.6858195087,2.0867504369
H,6.9318578831,2.3339232435,1.2035408378
H,5.2464586096,1.8882436214,-2.733292092
H,6.9068055283,2.9427224586,-1.2087159375
C,-4.6528397543,0.3094306852,-0.0198297878
C,-7.3757811837,1.0401823448,-0.1440912102

C,-5.0611211892,1.6237031195,0.2733698496
C,-5.636765854,-0.6313010828,-0.3762616523
C,-6.9840430875,-0.2698750175,-0.4371121482
C,-6.4083040711,1.9853479107,0.2112608755
H,-4.3189282911,2.3739606188,0.5292781262
H,-5.3503522111,-1.6578430639,-0.5845186429
H,-7.7280292189,-1.0141522404,-0.7057213981
H,-6.7005662203,3.007630509,0.4329012078
H,-8.4235693319,1.3213605209,-0.191594325
H,3.4897841448,-0.6923248708,1.8101747206

13e

Charge = 1 Multiplicity = 1
C,0.000000012,0.,-3.40713886
C,0.0000000023,0.,-0.552540631
C,-1.1936194689,-0.212968615,-2.6683853078
C,1.1936194879,0.212968615,-2.6683852997
C,1.1882068513,0.2248098906,-1.2854347524
C,-1.1882068417,-0.2248098906,-1.2854347604
H,-2.1247416997,-0.4242088381,-3.1803064225
H,2.1247417221,0.4242088381,-3.1803064081
H,2.1122152598,0.4416729211,-0.761648823
H,-2.1122152537,-0.4416729211,-0.7616488373
C,-0.0000000026,0.,0.9217880855
C,-0.0000000123,0.,3.7713209162
C,-1.1437544623,0.3829204138,1.6513616142
C,1.1437544521,-0.3829204138,1.6513616219
C,1.1404977021,-0.3879697269,3.0421196384
C,-1.1404977217,0.3879697269,3.0421196307
H,-2.0336462084,0.7198778067,1.1299343322
H,2.0336462017,-0.7198778067,1.129934346
H,2.0269906163,-0.7260652809,3.5690010378
H,-2.0269906394,0.7260652809,3.5690010241
C,-0.0000000173,0.,5.2555707303
C,-0.0000000268,0.,8.0783163809
C,-1.1516149466,-0.3656886028,5.9778422143
C,1.1516149072,0.3656886028,5.9778422221
C,1.1510597592,0.3669454001,7.3738620678
C,-1.1510598081,-0.3669454001,7.37386206
H,-2.0458606501,-0.6750656861,5.445382663
H,2.0458606142,0.6750656861,5.4453826768
H,2.0477849112,0.662141798,7.9108557208
H,-2.0477849637,-0.662141798,7.910855707
H,-0.0000000305,0.,9.1642227306
C,0.0000000169,0.,-4.8540734624
C,0.0000000266,0.,-7.744441594
C,1.216285376,-0.2101591966,-5.5901291686
C,-1.2162853373,0.2101591966,-5.5901291768
C,-1.2256765076,0.2177960587,-6.9501586967
C,1.2256765555,-0.2177960587,-6.9501586884

H,2.1369049874,-0.4034870156,-5.0547807888
H,-2.1369049523,0.4034870156,-5.0547808032
H,-2.1527317446,0.3992826264,-7.4855420302
H,2.1527317961,-0.3992826264,-7.4855420156
H,-0.1506548634,-0.8450122461,-8.4404853267
H,0.1506549213,0.8450122461,-8.4404853257

13f

Charge = 1 Multiplicity = 1
H,-1.2246067145,1.5812230306,-4.560081891
C,-1.3084675053,1.0261850113,-3.6298291074
C,-1.5461948718,-0.3730567928,-1.1864589667
C,-0.2706910485,0.1152673478,-3.2939818178
C,-2.4361003889,1.2651143601,-2.8199851025
C,-2.5543843937,0.6156642241,-1.6174425138
C,-0.3932983575,-0.553052077,-2.0910234398
H,-3.1834429502,1.9802565636,-3.1441384946
H,-3.4025610537,0.7908343001,-0.9629022875
H,0.3556227962,-1.2773371814,-1.7856820662
C,-1.1741297316,-0.2921961033,0.302339813
C,-0.5032467317,-0.0866157135,3.0408676132
C,-0.3261909999,0.7303642006,0.7488038568
C,-1.6808515955,-1.2126794493,1.2244661812
C,-1.347756361,-1.1096525945,2.5755380412
C,0.0014044247,0.8297863,2.0998611757
H,0.0735335863,1.4570434387,0.0465359518
H,-2.3296799354,-2.0193240612,0.8951376887
H,-1.7334027893,-1.8490697375,3.2700315424
H,0.6403380558,1.6434288729,2.4279474541
C,-0.1544177002,0.0227470188,4.4820309552
C,0.5054101841,0.2269300185,7.2180418955
C,1.1390081833,0.4017746879,4.8853612885
C,-1.1107660815,-0.2507718911,5.4770058822
C,-0.7844487374,-0.1495502902,6.8309247956
C,1.4656239185,0.5020506974,6.2393826799
H,1.9014329824,0.5967493592,4.1370309537
H,-2.1224890058,-0.522669327,5.1912243595
H,-1.5405631104,-0.3571795972,7.5824784866
H,2.4727250714,0.7875173929,6.5290255126
H,0.7595293379,0.3051904944,8.2709030007
C,0.8919524504,-0.0873301311,-4.1969000717
C,3.0919947485,-0.4831996966,-5.9003578864
C,0.718695627,-0.1129952639,-5.5913962081
C,2.1822213836,-0.2607071215,-3.6670805251
C,3.2738021167,-0.4564446882,-4.514260905
C,1.8120559365,-0.3122245075,-6.4359593329
H,-0.2721916586,-0.0025763722,-6.0221335632
H,2.3406232751,-0.2180008193,-2.5935814524
H,4.2659967288,-0.5785635862,-4.0910885374
H,1.6618526227,-0.3391046416,-7.5107062867

H,3.9420612636,-0.6347578849,-6.5583581019
H,-2.096002191,-1.3390677399,-1.3015906

14

Charge = 0 Multiplicity = 1
C,-0.7216064298,-0.1823762031,1.7956290728
C,-3.4579756014,-0.8491954785,1.7904078602
C,-1.5166637236,0.0320994978,0.6575518486
C,-1.3250026364,-0.7393739535,2.9372526993
C,-2.6815768482,-1.0677616886,2.9296025151
C,-2.8830503381,-0.2935757708,0.633517209
H,-1.0727896251,0.5030279202,-0.2140389362
H,-0.7287560473,-0.9358780817,3.822990206
H,-3.1330161674,-1.5125622045,3.8118224414
H,-4.5065298888,-1.1306519433,1.7921154515
C,0.7216064298,0.1823762031,1.7956290728
C,3.4579756014,0.8491954785,1.7904078602
C,1.3250026364,0.7393739535,2.9372526993
C,1.5166637236,-0.0320994978,0.6575518486
C,2.8830503381,0.2935757708,0.633517209
C,2.6815768482,1.0677616886,2.9296025151
H,0.7287560473,0.9358780817,3.822990206
H,1.0727896251,-0.5030279202,-0.2140389362
H,3.1330161674,1.5125622045,3.8118224414
H,4.5065298888,1.1306519433,1.7921154515
C,3.7014026035,0.0449600412,-0.5844860761
C,5.2547581859,-0.4275604078,-2.8963609209
C,5.0241010533,-0.4242640856,-0.4833509823
C,3.1740365209,0.2723821656,-1.8690964248
C,3.941358607,0.0384182855,-3.0124383753
C,5.7926352464,-0.6573894506,-1.626115015
H,5.4474896162,-0.6292416989,0.4954570502
H,2.1635141322,0.6560977591,-1.9738790054
H,3.5152057016,0.2286888207,-3.9933909633
H,6.8089751665,-1.0269924961,-1.5235150246
H,5.8520269311,-0.6093557917,-3.7850268003
C,-3.7014026035,-0.0449600412,-0.5844860761
C,-5.2547581859,0.4275604078,-2.8963609209
C,-3.1740365209,-0.2723821656,-1.8690964248
C,-5.0241010533,0.4242640856,-0.4833509823
C,-5.7926352464,0.6573894506,-1.626115015
C,-3.941358607,-0.0384182855,-3.0124383753
H,-2.1635141322,-0.6560977591,-1.9738790054
H,-5.4474896162,0.6292416989,0.4954570502
H,-6.8089751665,1.0269924961,-1.5235150246
H,-3.5152057016,-0.2286888207,-3.9933909633
H,-5.8520269311,0.6093557917,-3.7850268003

14a

Charge = 1 Multiplicity = 1
C,0.1180872874,2.0175557422,-3.445904281
C,0.0310404877,1.8943565183,-0.5790508966
C,0.3883478295,0.739715257,-2.7212268717
C,-0.1893682696,3.1973346957,-2.6085398483
C,-0.2285660704,3.1364465742,-1.2553242098
C,0.3311273522,0.7371841685,-1.3372693205
H,-0.3966310979,4.130490047,-3.1233797567
H,-0.4874533269,4.0167282677,-0.680921746
H,0.5850925749,-0.1679832218,-0.8024352677
C,-0.0025441657,1.8348554739,0.8830140129
C,-0.0764861943,1.7173005347,3.6851610912
C,-0.3067747518,0.6272340262,1.5498934857
C,0.2608664552,2.9889151009,1.6556664415
C,0.2337963324,2.9187867613,3.0443447826
C,-0.3582065394,0.5516864407,2.9461294705
H,-0.5688319292,-0.2524004192,0.9733820286
H,0.5272018621,3.9243063728,1.1778340739
H,0.464607776,3.8003439877,3.6333621987
H,-0.0837260189,1.6778289187,4.7697718897
C,0.7056218204,-0.4716152885,-3.4832273544
C,1.2944926846,-2.8085669837,-4.9486589599
C,1.2019301748,-0.3950021478,-4.8044580314
C,0.503300941,-1.7536763443,-2.9207514096
C,0.7914718322,-2.9052320693,-3.6464881075
C,1.4981025972,-1.5494817565,-5.523627352
H,1.3842621493,0.5662612859,-5.2708076462
H,0.0835531839,-1.8541712044,-1.9265988673
H,0.6157976215,-3.8787088239,-3.2002704522
H,1.89223821,-1.4672789019,-6.5313188795
H,1.522992111,-3.7076895197,-5.5122871391
C,-0.7054849654,-0.7222241756,3.630374595
C,-1.3683168962,-3.1352191114,4.9283380601
C,-0.2058485436,-1.9509427242,3.162966766
C,-1.5408720849,-0.7239537355,4.7618198768
C,-1.8700339078,-1.9196478995,5.4036209992
C,-0.5340306403,-3.1461645959,3.8062172691
H,0.4645936334,-1.970892895,2.3086955167
H,-1.9534553188,0.2109699444,5.129519508
H,-2.5235474682,-1.9012358747,6.2707852242
H,-0.1301842302,-4.0839070449,3.4360582806
H,-1.6232652547,-4.064519367,5.4288085015
H,-0.7082412521,1.865189902,-4.159681712
H,0.9633440697,2.2770240048,-4.103535783

14b

Charge = 1 Multiplicity = 1
C,-0.8159484707,-1.8208907059,0.0029993317
C,-3.6703680278,-1.8218131659,-0.3491114778
C,-1.569229236,-0.7948262703,0.5399803902

C,-1.5196349147,-2.8304275165,-0.7070213223
C,-2.9181255597,-2.842762283,-0.8728691028
C,-3.0421836903,-0.7431492463,0.436689187
H,-1.093381226,-0.001374886,1.1080871522
H,-0.9471619712,-3.6365574521,-1.1574355329
H,-3.3815317092,-3.6474396239,-1.4321591346
H,-4.7490090465,-1.7985955864,-0.4686324428
C,0.6629709444,-1.8751551104,0.1423243442
C,3.450199516,-1.9632349731,0.4029114476
C,1.3114709368,-3.0996017684,0.3718853282
C,1.4259347131,-0.7018450074,0.0428371765
C,2.8241546376,-0.725487785,0.1697968106
C,2.7000704708,-3.1354132921,0.5025138115
H,0.7392518124,-4.0164426408,0.475973851
H,0.9333342175,0.2384958287,-0.1846995447
H,3.1979242884,-4.0801212764,0.6975233213
H,4.5278229372,-2.0060679273,0.5253664625
C,3.6198652767,0.5252582971,0.047378982
C,5.1316067283,2.8962014612,-0.1889374506
C,4.8554113197,0.5284469094,-0.625234332
C,3.1572437119,1.7334112392,0.5997956455
C,3.9053414231,2.9069133961,0.4829947169
C,5.6034439088,1.7018435644,-0.7423529252
H,5.2235356809,-0.3868094015,-1.0791249772
H,2.2184707626,1.7513447399,1.145582045
H,3.5340034818,3.8269986484,0.9248580004
H,6.5509557026,1.6837933585,-1.2729494867
H,5.7136431694,3.8084635432,-0.2795671542
C,-3.6122475183,0.6618348073,0.1873527778
C,-4.6114931774,3.2319720355,-0.2934900351
C,-3.5357929611,1.2158325917,-1.0984636418
C,-4.188683098,1.3959980449,1.2295278259
C,-4.6869116922,2.679763062,0.9877479563
C,-4.0358890685,2.4980117011,-1.3355195787
H,-3.0901137251,0.6514569833,-1.9129394655
H,-4.2520278515,0.9722269604,2.2277717997
H,-5.1343032851,3.2431250116,1.8007060881
H,-3.9750054926,2.9212126276,-2.3334079017
H,-4.9995740176,4.2284888362,-0.480261038
H,-3.3562705901,-1.0103483791,1.4767874428

14c

Charge = 1 Multiplicity = 1
H,-0.380863155,2.2132724755,-4.3607182368
C,-0.4403005754,1.8380362546,-3.3428369763
C,-0.7009102471,0.8442141482,-0.7107117084
C,0.2027710569,0.6076074858,-3.0409441656
C,-1.1675175346,2.5936075179,-2.4029515902
C,-1.286576053,2.1380016704,-1.1139855852
C,0.0960185115,0.149402423,-1.7417708562

H,-1.6262947227,3.5269844495,-2.7088974844
H,-1.8497094886,2.6931132902,-0.3701524215
H,0.5839157338,-0.7728851896,-1.4413557786
C,-0.0956472918,0.8161873764,0.6988653416
C,1.0045028671,0.8111796222,3.263760594
C,-0.7476611978,0.152535303,1.7409293599
C,1.1137871329,1.4824874385,0.9379524487
C,1.6570897833,1.474833441,2.2235677604
C,-0.2087586189,0.1378041568,3.0401820799
H,-1.6957760695,-0.3434118764,1.553679863
H,1.6272857297,2.0009534515,0.1334650103
H,2.5981220752,1.9820308037,2.4127345482
H,1.4524866246,0.7972677019,4.2523339446
C,0.967866582,-0.1314088299,-4.0780511408
C,2.4199694247,-1.5317529623,-6.0334775427
C,1.7636271961,0.5608787933,-5.0068147611
C,0.9084041961,-1.5341616525,-4.1414272696
C,1.6295983522,-2.2281711549,-5.1141398054
C,2.4857280976,-0.1363363954,-5.9766390505
H,1.8428081874,1.6430558278,-4.9601568634
H,0.27868464,-2.0846814449,-3.4485948186
H,1.5666809654,-3.3109744195,-5.1582818898
H,3.1038583842,0.4105167695,-6.6817774009
H,2.9804150758,-2.0727913651,-6.789620724
C,-0.9084278923,-0.5721251278,4.1440424659
C,-2.2321768559,-1.917668362,6.2410618245
C,-1.525305202,-1.8173059217,3.9253873648
C,-0.9673363237,-0.0143574243,5.4340984944
C,-1.6231941835,-0.6801129333,6.4717754411
C,-2.180044971,-2.483636699,4.9634834345
H,-1.471845175,-2.2798592208,2.9441809809
H,-0.5165267626,0.955541555,5.6223393424
H,-1.6640441654,-0.2280563971,7.4584771912
H,-2.6416253894,-3.4486783598,4.7754751517
H,-2.7412322454,-2.4357289169,7.0483181142
H,-1.6118129261,0.1945927674,-0.6628049858

15

Charge = 0 Multiplicity = 1
C,0.0555763801,0.9605678028,-0.8030447307
C,0.1503091263,0.9401621605,2.0451316944
C,0.9869250217,0.165813163,-0.1131200243
C,-0.8220535146,1.7495510611,-0.0406886914
C,-0.7790761119,1.7368514525,1.3536736081
C,1.0349784752,0.1580294683,1.2797488293
H,1.6942375348,-0.4391077547,-0.6717870484
H,-1.5636587873,2.3635929357,-0.5439377538
H,-1.4944179711,2.3357292017,1.9095019158
H,1.7874265177,-0.4461727952,1.7779305658
C,0.0306429824,1.0209863324,-2.293906701

C,0.1148035724,1.2951314992,-5.103262466
C,0.1163410543,2.2857960774,-2.904768831
C,-0.0364564201,-0.1308148223,-3.1189220241
C,0.0177110799,0.0338739053,-4.5150824921
C,0.155934497,2.4309258166,-4.2915708467
H,0.1818246506,3.1668862829,-2.2729665266
H,-0.0468941882,-0.8473822721,-5.1467489803
H,0.2294450482,3.4212419032,-4.7313259647
H,0.1468843103,1.3882340513,-6.1848514614
C,-0.2089288145,-1.5148430762,-2.5838010711
C,-0.580974872,-4.1594977176,-1.6695828237
C,0.6559547795,-2.5463117431,-2.9888199204
C,-1.2682444532,-1.8349786417,-1.7165286153
C,-1.4535365094,-3.143337427,-1.2651646087
C,0.4748016413,-3.8556755071,-2.5339706784
H,1.4843261862,-2.3182070052,-3.6538455609
H,-1.9560521442,-1.0560953889,-1.4021470456
H,-2.2827430338,-3.3696799249,-0.6008026065
H,1.160112794,-4.6356657466,-2.8535808301
H,-0.7244918727,-5.1765773343,-1.316590642
C,0.1980533757,0.9265893185,3.5310136777
C,0.2852980685,0.8997649896,6.355737555
C,0.461374507,-0.2628489337,4.2359513482
C,-0.0200769784,2.1023672845,4.2733362373
C,0.0220049095,2.0894008344,5.6692130985
C,0.5053183488,-0.2763608973,5.6318233292
H,0.610862615,-1.1889514831,3.6888170116
H,-0.2025064629,3.0387198191,3.7543478559
H,-0.1421795416,3.0114069854,6.2197565083
H,0.7025287694,-1.2085590546,6.1536150883
H,0.31861542,0.8894451603,7.4412206421

15a

Charge = 1 Multiplicity = 1
C,0.0297620473,0.5638077999,2.5657476476
C,0.188839086,0.3690351423,5.3714931839
C,-0.0207666459,-0.7119041201,3.1870521027
C,0.1271684277,1.7145607516,3.3729394504
C,0.210039737,1.6252858824,4.7607285038
C,0.0691457067,-0.778846675,4.5877939108
H,0.1808208742,2.6893193992,2.8979985299
H,0.2999354675,2.5277897005,5.3571687574
H,0.0130723626,-1.7513132421,5.0674562453
H,0.2510075984,0.2818686666,6.4519669973
C,0.043446084,0.7526886765,1.0937729322
C,0.140182843,1.2217333266,-1.7148012404
C,-0.755375805,1.7497109519,0.4940279715
C,0.8963358211,-0.0018028498,0.2593430247
C,0.9411164065,0.2199693496,-1.1056163831
C,-0.7068943956,1.9844894537,-0.8699789479

H,-1.439344014,2.3275194152,1.1065190156
H,1.5441572015,-0.7535518511,0.6950677743
H,1.6402116473,-0.3584152396,-1.6971781586
H,-1.3681189143,2.7349032232,-1.2861651924
C,0.1897922122,1.459551029,-3.143393651
C,0.2998446089,1.93833672,-5.9910381418
C,-0.2760482904,2.6996128664,-3.6996389245
C,0.7081602802,0.4615745724,-4.0377168788
C,0.7604487191,0.6760179515,-5.3800585903
C,-0.2244582704,2.9363586713,-5.0382206805
H,-0.6422479734,3.4762990684,-3.0409238312
H,1.0328659073,-0.4925657144,-3.6432107822
H,1.1411991221,-0.0986919634,-6.0387616313
H,-0.5645539309,3.8883987243,-5.4342649522
C,-0.2304853016,-1.9826991454,2.4335840821
C,-0.6672224898,-4.423037795,1.0902989131
C,-1.3221766875,-2.1376656433,1.5611138692
C,0.6325717873,-3.0752717021,2.6285831137
C,0.4191226781,-4.2832128267,1.9595273136
C,-1.5392914044,-3.3466444038,0.8968398911
H,-2.0141427955,-1.3133965753,1.4160846506
H,1.4835035901,-2.9725566425,3.2960510143
H,1.1020818006,-5.1129610208,2.1169387755
H,-2.3937904612,-3.4498267123,0.2342750615
H,-0.8360900872,-5.3629431825,0.5731990643
H,1.1154751847,2.383364524,-6.5893167423
H,-0.4593010245,1.7251112786,-6.7652872079

15b

Charge = 1 Multiplicity = 1
C,0.3953526277,0.4743554745,2.5113555213
C,0.2350308318,0.284802795,5.3118939585
C,-0.2513438735,-0.6399701885,3.101276334
C,0.9598603486,1.4621579949,3.3367504022
C,0.8871966984,1.3719980016,4.7264153914
C,-0.3243433122,-0.7049509619,4.5032199186
H,1.4342022895,2.3263539623,2.8808153157
H,1.3251223663,2.1504901402,5.3432047151
H,-0.8057984978,-1.5634218465,4.9615654422
H,0.1673413907,0.2015932058,6.3922981108
C,0.4706041468,0.6670617383,1.0354408669
C,0.4516039646,1.107809121,-1.8548701419
C,-0.6987078394,0.5600861215,0.2169947878
C,1.6329470551,1.0025013081,0.3973693583
C,1.7041694003,1.2497053428,-1.0585288909
C,-0.7114770526,0.7674563382,-1.1591264567
H,-1.6389996354,0.3084759411,0.6975111415
H,2.5630261623,1.0898650261,0.9507469608
H,-1.6505199897,0.6361424779,-1.6816454649

C,0.4628275616,1.3389603973,-3.293614766
C,0.4778115086,1.8066364905,-6.072979589
C,-0.7385084076,1.6144057341,-3.992226375
C,1.6720110646,1.3104926213,-4.0296093012
C,1.6761304285,1.5355910132,-5.4013286471
C,-0.7271794665,1.8493718577,-5.3625276162
H,-1.6797128041,1.6820350963,-3.459901809
H,2.6116921827,1.0868903303,-3.5382550882
H,2.6114083672,1.4948150077,-5.9497511317
H,-1.6561518528,2.0719985888,-5.8769527374
H,0.4839035323,1.9843098127,-7.143777641
C,-0.8245359858,-1.7645148116,2.3039810048
C,-1.9016092382,-3.9412918198,0.8702542268
C,-2.1528675797,-2.1773071888,2.5130937203
C,-0.0438033216,-2.4671172796,1.3680413886
C,-0.5771025768,-3.5465267339,0.6592044534
C,-2.6873200527,-3.2534965424,1.8007365328
H,-2.7719073005,-1.6444471241,3.2292154507
H,0.9921479943,-2.1823207373,1.2115065086
H,0.0453331219,-4.083145153,-0.0508826498
H,-3.7170152988,-3.5532715786,1.97206507
H,-2.3161677236,-4.7797781823,0.3187137432
H,2.1175335902,2.2608016567,-1.2176627458
H,2.4978149657,0.6043967325,-1.4700293024

15c

Charge = 1 Multiplicity = 1
C,-0.0497848056,0.6116137914,2.2719997722
C,0.0994868974,0.4192086868,5.1423248355
C,-0.0261605237,-0.6970406243,2.8975942747
C,-0.0893675764,1.7851101782,3.0979697702
C,-0.003631851,1.714782483,4.453469672
C,0.05300558,-0.7624733685,4.2650971413
H,-0.1089027801,2.7552428117,2.6169577946
H,0.018905485,2.621577018,5.0497854339
H,0.0512049789,-1.7354929174,4.7474426469
C,-0.0086193966,0.8060851312,0.8295950788
C,0.1269781657,1.2822979573,-1.9755498831
C,-0.692321827,1.8965595092,0.2352987123
C,0.752869116,-0.033826715,-0.0196570042
C,0.8343718721,0.2158159633,-1.3797804541
C,-0.6438708686,2.1115665308,-1.132465354
H,-1.3171374162,2.5425950987,0.8421803069
H,1.3266211599,-0.8515358122,0.3971705774
H,1.4734572093,-0.4131021386,-1.9895634847
H,-1.2302874785,2.9184851939,-1.5579443204
C,0.1913815748,1.5216344185,-3.4340067794
C,0.3078598727,1.9748937068,-6.2140543064
C,0.1299734895,2.8291777697,-3.9547617657
C,0.3128357486,0.4459176017,-4.3356812555

C,0.3656511152,0.6709458925,-5.7112936305
C,0.1923891525,3.0528067684,-5.3302147518
H,0.061686465,3.6773409399,-3.2809059448
H,0.3365837533,-0.5732293614,-3.9626646411
H,0.4456564271,-0.1721889018,-6.3907349876
H,0.1566578885,4.0689213255,-5.7112549552
H,0.35240519,2.1495043401,-7.2847652747
C,-0.1595313423,-1.9788379579,2.1378482008
C,-0.466213397,-4.4453430299,0.8262000146
C,-1.2830769992,-2.219992211,1.3299191095
C,0.803237463,-2.9890385039,2.2868713063
C,0.6523069387,-4.214196025,1.6311152531
C,-1.4350553893,-3.4463313012,0.6812128469
H,-2.0433140898,-1.4531093543,1.2154713968
H,1.6789430492,-2.8116273781,2.9046657012
H,1.4091724761,-4.983645226,1.7493536291
H,-2.3118256122,-3.622676367,0.0654882469
H,-0.5850513652,-5.397333645,0.3178386085
H,1.013743597,0.3929839447,5.7643035666
H,-0.6932318367,0.3359536274,5.908124656

15d

Charge = 1 Multiplicity = 1
H,0.0126851474,0.9173307403,-6.1043305142
C,-0.0280035683,0.8774409667,-5.0218864535
C,-0.098647365,0.9217715927,-2.1270773186
C,-0.5732785301,-0.1844236453,-4.371154289
C,0.4883597814,1.955994517,-4.2520809539
C,0.4507253531,1.9797285332,-2.861725916
C,-0.615004203,-0.279993055,-2.881437592
H,-0.9864861743,-1.0262491359,-4.9178696921
H,0.9127473135,2.811034694,-4.7704856868
H,0.8196293788,2.863429719,-2.3565360302
C,-0.1785600663,0.9622032858,-0.681877605
C,-0.3189770857,1.0492615227,2.1676570617
C,-1.0734414046,0.1312113344,0.0394199159
C,0.6465484052,1.8369194871,0.0728727112
C,0.5822901428,1.8705709588,1.4546675235
C,-1.1463849081,0.1816976094,1.4208104965
H,-1.7530796484,-0.5295799144,-0.4845960999
H,1.3842207226,2.456294242,-0.4229687563
H,1.263134649,2.5187437112,1.9950431671
H,-1.8769142234,-0.4375474895,1.9291561533
C,-0.3938332042,1.0982210004,3.6428245289
C,-0.5291911028,1.1907585452,6.4571796465
C,-0.7084050952,-0.0555164488,4.388501653
C,-0.1500086398,2.2997382402,4.337997522
C,-0.2217341963,2.3454566112,5.7299138673
C,-0.7700729868,-0.0097950081,5.7809196092
H,-0.8761660966,-0.9998352983,3.8806771056

H,0.0682338886,3.2093611778,3.787676199
H,-0.0431440355,3.2836468182,6.2463881064
H,-1.0000096629,-0.9127295974,6.33829782
H,-0.5809337926,1.226224441,7.5411145528
C,0.0834346268,-1.603473772,-2.4813516221
C,1.3422771845,-4.0287331705,-1.8547684948
C,-0.6779809358,-2.7616735611,-2.2833980757
C,1.4797138068,-1.6668133953,-2.3803552008
C,2.1043815816,-2.8751603274,-2.0626029745
C,-0.0492979511,-3.9696155026,-1.9679021643
H,-1.76051886,-2.7269484272,-2.3712815553
H,2.0842416395,-0.7790385274,-2.5420193346
H,3.1862252354,-2.9126152717,-1.9790614171
H,-0.6496140555,-4.8601636143,-1.8088187302
H,1.8296499289,-4.9665286211,-1.6067195464
H,-1.6788108731,-0.4006058649,-2.6188113865

15e

Charge = 1 Multiplicity = 1
C,1.0619726448,0.251964436,-0.9482720864
C,1.5796084941,0.9397328317,1.8233704149
C,0.1964006266,1.1556030331,-0.2475650907
C,2.1750116378,-0.3213793638,-0.2480582253
C,2.4082683744,-0.028975488,1.0604365069
C,0.4359690554,1.4884245211,1.0500706597
H,-0.6322200754,1.6094697326,-0.7753404233
H,2.8051954919,-1.0397773841,-0.758646804
H,3.2313015687,-0.5035918801,1.5864091691
H,-0.2021270948,2.2089702718,1.5527668284
C,0.8862493,-0.0193764486,-2.3707822474
C,0.7380602184,-0.1916427535,-5.1729934636
C,2.0601451682,-0.0844171537,-3.1644066243
C,-0.3899791549,-0.110395421,-3.0086529891
C,-0.4287761655,-0.1837560531,-4.4074256869
C,1.9896613255,-0.1525438274,-4.5486782008
H,3.0317550659,-0.0060697825,-2.6897975383
H,-1.391185187,-0.2790037361,-4.8993461293
H,2.9003997898,-0.1656113944,-5.1377660586
H,0.6699317025,-0.2519968034,-6.2546468399
C,-1.6640095666,-0.2460004271,-2.2556532188
C,-4.1029944794,-0.5537613927,-0.8842728618
C,-2.7811598202,0.5389764656,-2.5958768509
C,-1.7945558128,-1.1999694995,-1.2294572507
C,-3.0049980966,-1.3528553716,-0.5513385163
C,-3.9872258008,0.3915979912,-1.9092473277
H,-2.6962766473,1.2824357205,-3.3829123905
H,-0.9552648416,-1.8430547944,-0.9813703744
H,-3.0927238227,-2.1019762485,0.2297762892
H,-4.8353408643,1.0160062775,-2.1733019771
H,-5.0435927612,-0.6714691154,-0.3549666762

C,1.1908065739,0.4317147809,3.2217278914
C,0.4850857875,-0.5272850626,5.7589852324
C,0.2617898235,-0.6107263649,3.3482866228
C,1.7645918084,0.9903169222,4.3693024747
C,1.4118469186,0.5110240566,5.6348620327
C,-0.0893991915,-1.087213387,4.6131450848
H,-0.18717517,-1.0499041647,2.4616299402
H,2.485805075,1.7980930625,4.2799882578
H,1.8610354988,0.9512999101,6.5198740381
H,-0.8105538247,-1.8940228997,4.7023403706
H,0.2110300548,-0.8981652403,6.7418942473
H,2.2476360728,1.8093113646,1.9959101409

15f

Charge = 1 Multiplicity = 1
C,0.2415299612,-0.1424707024,1.3545872487
C,-0.5164788963,0.1039922416,4.025392815
C,0.5211773959,-1.2109587227,2.2519221789
C,-0.4007506662,1.0287209936,1.80379775
C,-0.7796884914,1.1500101588,3.1301108713
C,0.1238093905,-1.0550012028,3.5863862424
H,-0.6033411081,1.8380093273,1.112593278
H,-1.2771187898,2.0527153045,3.46753081
H,0.324390368,-1.8613050666,4.2838783693
H,-0.809950196,0.1935155805,5.0663856198
C,1.439284562,0.0303177528,0.0155409691
C,-0.4281995764,1.4554074881,-1.6185236102
C,1.7457258283,1.3552960299,-0.458209648
C,0.1650510653,-0.5486201477,-0.3215337424
C,-0.7501579765,0.203300549,-1.154512384
C,0.8539859319,2.0222041003,-1.2459820213
H,2.715196619,1.7820954503,-0.2275604297
H,-1.6959327982,-0.2613203557,-1.4088662723
H,1.1277456079,2.9917115607,-1.6486153975
H,2.2518365679,-0.6064424451,0.3434879066
H,0.0581373176,-1.6273537588,-0.3215811629
C,1.2107619808,-2.475877872,1.8514094334
C,2.502336776,-4.8800868726,1.1871915874
C,0.4758257856,-3.5613648578,1.3469339023
C,2.5967513786,-2.6116000405,2.0335228716
C,3.237906182,-3.8077229756,1.6997348526
C,1.1204719143,-4.756231308,1.0161289096
H,-0.6009399771,-3.4780403694,1.2293142335
H,3.1703242854,-1.7866479371,2.4472899778
H,4.3095488185,-3.9011366104,1.8466030815
H,0.5406283581,-5.5896428711,0.6315205272
H,3.0014987385,-5.8093559913,0.9304685315
C,-1.3367795526,2.2205064193,-2.5069164366
C,-3.0824846986,3.6760919535,-4.1688625728
C,-1.4369152518,3.6194530775,-2.3930990159

C,-2.121663842,1.5642031439,-3.473038997
C,-2.9849632774,2.2865445311,-4.2969650996
C,-2.3069701419,4.3393470516,-3.2136534487
H,-0.8557918051,4.1490178067,-1.6442034712
H,-2.0367953945,0.4894552198,-3.6000883806
H,-3.5743283395,1.7652029303,-5.0450533118
H,-2.3800560953,5.4169510581,-3.1035468062
H,-3.7546171487,4.2371103086,-4.8109195787

15g

Charge = 1 Multiplicity = 1
C,0.5849042543,-0.4086939072,2.4195815643
C,-1.2971777282,-0.2357072087,4.5406119371
C,0.1439096112,-1.5967013877,3.2039929191
C,0.0256468726,0.9196612239,2.8586757508
C,-0.9080529633,0.9384404321,3.8955380056
C,-0.7731235669,-1.5118483454,4.2070870369
H,0.5793947672,-2.5498181901,2.9204718484
H,-1.3173885984,1.8772884899,4.2471552952
H,-1.0988116907,-2.3904627705,4.7517037013
H,-2.0184323549,-0.1632146314,5.3497374828
C,0.3365387548,-0.7150980028,0.92230634
C,-0.1277472244,-1.3702763268,-1.7908190032
C,-0.9440916787,-0.5836577552,0.3704137045
C,1.3790771761,-1.1896478533,0.1192624404
C,1.1492332375,-1.5082086734,-1.219197258
C,-1.1684678164,-0.9022964352,-0.9680121531
H,-1.7743210824,-0.2364216061,0.9788303327
H,2.3790676824,-1.3020454991,0.5285783346
H,1.9809518938,-1.8453343901,-1.8295476352
H,-2.1722018202,-0.8066356217,-1.3698570838
C,-0.3706737971,-1.7091538537,-3.2176992365
C,-0.8327808389,-2.3546692586,-5.9256406138
C,-1.23854404,-0.9318456664,-4.0062806052
C,0.2611409674,-2.8163256561,-3.8131636913
C,0.0320809576,-3.1360615258,-5.1529968046
C,-1.466682483,-1.2509036446,-5.3465674254
H,-1.7202234444,-0.058939325,-3.5759122618
H,0.9177421283,-3.4453911934,-3.2195028106
H,0.5232415626,-4.0004600587,-5.5903212652
H,-2.1336840273,-0.6321963455,-5.9398359306
H,-1.0106003992,-2.603049854,-6.9677143503
C,0.4624967175,2.1441906024,2.2039317226
C,1.288799784,4.5203495506,0.93727933
C,-0.3677941371,3.2929611247,2.199725578
C,1.7174673834,2.221994618,1.5528448619
C,2.1265070214,3.3986666648,0.9363275812
C,0.0393165304,4.461122922,1.5663257437
H,-1.3514769161,3.257175106,2.6534730567
H,2.3937888437,1.3756284533,1.5604845663

H,3.0998447978,3.4453277824,0.4593484728
H,-0.6177198725,5.3244599144,1.5567319611
H,1.6087572177,5.4364842337,0.4509458211
H,1.6800883884,-0.3586858918,2.5417029996

16

Charge = 0 Multiplicity = 1
C,2.1111810012,-1.1051321154,0.2271949174
C,4.6158411896,-0.6124542367,1.4336594705
C,2.6020551939,0.2220375588,0.3191083943
C,2.9033460019,-2.1554330619,0.7257025586
C,4.1428669494,-1.9217141448,1.3216414576
C,3.8489084554,0.4400050143,0.9334607156
H,2.5210847991,-3.1704213172,0.6668342258
H,4.7268419044,-2.7541666979,1.7030875253
H,4.23102824,1.4550799153,0.9916979345
H,5.5776508062,-0.4107545515,1.8960006377
C,0.7700055323,-1.4462392528,-0.3359691631
C,-1.763749856,-2.1801247178,-1.3140019413
C,0.6468203288,-2.4290301094,-1.3321240171
C,-0.3963093644,-0.8458148442,0.1626459159
C,-1.6708098125,-1.1986759779,-0.310170642
C,-0.6130784588,-2.7879355325,-1.81724212
H,1.537751505,-2.9011975944,-1.7359288618
H,-0.3099137258,-0.1109088359,0.9566099203
H,-0.6966815695,-3.5365997112,-2.5998980853
H,-2.7353431118,-2.4529733968,-1.7145518355
C,-2.8921283649,-0.5535524712,0.24401122
C,-5.2113039703,0.6703084388,1.2952510106
C,-4.0667257927,-1.2971776129,0.4619532814
C,-2.9047375123,0.8167702542,0.5636723518
C,-4.0510212007,1.4218338682,1.0840085058
C,-5.2137209946,-0.6924701012,0.9812743184
H,-4.0766686341,-2.3610626106,0.2438159122
H,-2.0183329181,1.4176301233,0.3839242892
H,-4.0393625837,2.4829806664,1.3163590134
H,-6.1063397455,-1.2887053402,1.1482355679
H,-6.1027258499,1.1408547934,1.6996351182
C,1.8820524001,1.4043231243,-0.2425696551
C,0.6206068346,3.691671521,-1.3122101498
C,1.4765152679,1.440115504,-1.5882633252
C,1.6483457596,2.5381537897,0.554801276
C,1.020947112,3.6703950728,0.0269641657
C,0.8538239323,2.5721426067,-2.1182525579
H,1.6587767318,0.5798337391,-2.2248168221
H,1.9485478505,2.5274643379,1.598910796
H,0.8435709961,4.5327904066,0.6634135604
H,0.5537276588,2.5807160369,-3.1622925263
H,0.1339620448,4.5708838825,-1.7243644079

16a

Charge = 1 Multiplicity = 1
C,0.8505496468,-1.4884852435,-0.2912310972
C,-1.727555724,-2.4096618731,-1.139143121
C,-0.3194423774,-0.814051825,0.1280080281
C,0.7433268449,-2.6498596768,-1.1317518818
C,-0.4739277094,-3.0823318838,-1.5431194535
C,-1.5900643967,-1.225941634,-0.2387909927
H,-0.2035684267,0.0156251474,0.8118253038
H,1.6448946313,-3.1509812414,-1.4630914697
H,-0.5608112418,-3.941981546,-2.2006155381
C,2.1730057331,-1.0705124754,0.1914520097
C,4.638316626,-0.4548878912,1.3957539246
C,3.0616955132,-2.0918720235,0.6013718149
C,2.5620869839,0.2926370897,0.3449025765
C,3.7948645495,0.5660265551,0.9556572967
C,4.2743042102,-1.7919081568,1.2106023641
H,2.765800834,-3.1301814353,0.4972946611
H,4.1110981362,1.5995701471,1.0527418434
H,4.9258487398,-2.5929200701,1.5436496353
H,5.58623708,-0.2057319492,1.8624453311
C,1.7755706366,1.4348399575,-0.1977018815
C,0.371658145,3.6441122735,-1.2383667419
C,1.4981468214,2.5531338695,0.6089767079
C,1.3495036917,1.4479015595,-1.5382917086
C,0.6543793171,2.543710001,-2.05364961
C,0.7965761232,3.6453946219,0.0941511947
H,1.8171952911,2.5584997554,1.6471123548
H,1.5867447546,0.6098083021,-2.1869399962
H,0.3421282842,2.5410801619,-3.0936776088
H,0.5816664849,4.4957587693,0.734416541
H,-0.1695869916,4.4957044541,-1.6393395671
C,-2.7903734026,-0.5273051617,0.2293493324
C,-5.0970163403,0.8370039836,1.1071896847
C,-2.7223023118,0.8232599786,0.6445925746
C,-4.0480613851,-1.1714427999,0.2585952148
C,-5.1844348831,-0.4982124793,0.6982295523
C,-3.8626530807,1.4951301145,1.0740182065
H,-1.7836740837,1.3633590855,0.596687295
H,-4.142425183,-2.2101833494,-0.0368048673
H,-6.1376368581,-1.0161214299,0.7263799593
H,-3.7907891995,2.5351401173,1.3755821839
H,-5.9847224563,1.3613827206,1.4468982188
H,-2.2777719904,-2.1149419861,-2.0485656288
H,-2.3909943067,-3.1655622936,-0.689423466

16b

Charge = 1 Multiplicity = 1
C,0.5589757909,-1.4107159174,-0.507377889

C,-1.985630773,-1.7288373792,-1.6304758024
C,-0.5567606453,-0.7919229264,0.0737431988
C,0.3902367212,-2.2004641344,-1.6538950838
C,-0.8814287833,-2.3514947517,-2.2119511771
C,-1.8412175788,-0.9413862872,-0.4730094136
H,-0.4303206747,-0.211264688,0.9823007628
H,1.2486461215,-2.6802591069,-2.1147669888
H,-1.0085525138,-2.9492906077,-3.1093064974
H,-2.9641787728,-1.838684089,-2.0871860571
C,1.9037866667,-1.3065624309,0.1391139074
C,4.3883589866,-1.1825704606,1.5711797862
C,2.5132986421,-2.4625075911,0.5593193969
C,2.5685215894,-0.04823876,0.4030265327
C,3.7840226834,-0.0332760158,1.1618391705
C,3.7983194749,-2.4947820413,1.2736587957
H,2.0188273106,-3.4136144053,0.3854474599
H,4.2647381877,0.9191697332,1.3484963974
H,5.334461664,-1.1480314279,2.1022173394
C,2.0715080817,1.2349211831,-0.0945676122
C,1.2303879304,3.7383284369,-1.0597566255
C,2.2022461535,2.3980987479,0.7015356277
C,1.5155318854,1.3580935474,-1.3894489495
C,1.1214684166,2.6022141757,-1.8704930967
C,1.7627887131,3.6308391764,0.2303684678
H,2.6036209408,2.3271164312,1.7067054366
H,1.4312315224,0.4889753925,-2.0297336906
H,0.7241221317,2.6865490697,-2.8766361505
H,1.8395682151,4.5077883185,0.8646476594
H,0.9029469491,4.7038106935,-1.4327136603
C,-3.0180315134,-0.2885268023,0.1612121666
C,-5.2531190272,0.9482845795,1.3613734113
C,-2.9228317363,1.0082001682,0.6984464907
C,-4.2557408252,-0.9529866699,0.2370890679
C,-5.3616328471,-0.3412105974,0.8313392038
C,-4.028851356,1.6203356273,1.2921247422
H,-1.9849078624,1.5514813807,0.6309984486
H,-4.3489788051,-1.9626627723,-0.151672181
H,-6.3056439711,-0.8755394761,0.8863909202
H,-3.9357807138,2.625803471,1.6923026853
H,-6.1132976847,1.4242961349,1.8226424085
H,4.5237918294,-3.1161433735,0.7153442776
H,3.6848888559,-3.0778064251,2.2062033537

16c

Charge = 1 Multiplicity = 1
C,1.8708693878,-1.2138127813,-0.3671134624
C,4.4145055935,-0.6700955477,-1.6275418053
C,2.129864967,0.1285422312,-0.9981996143
C,2.8948421705,-2.1612490685,-0.3686952816
C,4.1231855743,-1.8963377712,-0.9750669666

C,3.45641492,0.2975387368,-1.6548947701
H,2.733739753,-3.1417040674,0.0622964188
H,4.8810704441,-2.6746183626,-0.9639601929
H,3.6333640258,1.2480257382,-2.1485354046
H,5.3808988454,-0.5221559291,-2.0954392439
C,0.5719050596,-1.5017383004,0.2266098586
C,-1.9228251177,-2.0406671998,1.3931320306
C,0.4593683496,-2.4415472206,1.2793478746
C,-0.5959646964,-0.8434673896,-0.2197275461
C,-1.8498564299,-1.1084658006,0.3394743257
C,-0.7781620736,-2.6919304976,1.8596968877
H,1.340043937,-2.9356623321,1.6727449974
H,-0.5359185795,-0.1282787196,-1.0311346896
H,-0.8565494275,-3.4017433019,2.6765338239
H,-2.885991103,-2.274134003,1.8354980627
C,-3.0712749636,-0.4284517971,-0.1682531829
C,-5.3857974158,0.8636000464,-1.1258188247
C,-4.0624022752,0.0225263538,0.7215307238
C,-3.2613475148,-0.2227496096,-1.5462821206
C,-4.4084410705,0.4170525139,-2.0205068337
C,-5.2082772483,0.6638820609,0.2467579322
H,-3.927203414,-0.1073573286,1.7912611459
H,-2.5221533135,-0.5872778871,-2.253812907
H,-4.5421650318,0.5576822587,-3.0890375103
H,-5.9583154539,1.0131327669,0.9501508127
H,-6.2778226393,1.3609100852,-1.4946103681
C,1.9194677198,1.3152386869,-0.0232674858
C,1.597530508,3.4984621908,1.702089964
C,1.1716290085,2.4265741125,-0.4286392717
C,2.5206440168,1.305450052,1.2423131144
C,2.3541297764,2.3930544357,2.1027663998
C,1.0092988353,3.5138465784,0.4346662871
H,0.7114414307,2.4481675207,-1.4127215302
H,3.1122779199,0.4535749332,1.5644889035
H,2.8169002488,2.3743935178,3.0846808691
H,0.4203261107,4.3679547796,0.114941859
H,1.4676752298,4.3417235624,2.3733050929
H,1.3818173758,0.2624248533,-1.7977177218

16d

Charge = 1 Multiplicity = 1
C,2.0980174635,-0.9520268392,0.2771778771
C,4.6847105866,-0.6390874363,1.3566465449
C,2.622338493,0.348880543,0.4842122916
C,2.8951376169,-2.0657304354,0.5985191486
C,4.1758014414,-1.9198801083,1.1310844762
C,3.9115420977,0.4757782904,1.0323294089
H,2.487028188,-3.0616503702,0.4530711689
H,4.7647040533,-2.7988536482,1.3753444091

H,4.3205862667,1.4712664525,1.1776705302
H,5.6796518089,-0.5064881002,1.7710014143
C,0.7165503107,-1.2085679743,-0.2279932277
C,-1.8963883849,-1.7955705172,-1.1180536778
C,0.5009509654,-2.0914380746,-1.2982863979
C,-0.4021141008,-0.6269629805,0.3932837468
C,-1.6957019419,-0.9160005981,-0.0450261938
C,-0.7926317106,-2.380385214,-1.7392384723
H,1.3520037339,-2.5453105288,-1.7967094908
H,-0.2523671474,0.0468546141,1.231065981
H,-0.940848787,-3.0604781679,-2.5723521221
H,-2.9004844172,-2.0246579678,-1.4633029069
C,-2.9148801398,-0.2696485882,0.6320087393
C,-5.7275790196,0.0281486036,0.4178858915
C,-3.8578607209,-1.2273472944,1.2483835474
C,-3.5349315877,0.8241091208,-0.1481719155
C,-4.8971170542,0.9698483742,-0.2260537111
C,-5.2165653215,-1.0601957371,1.1568451117
H,-3.4339795335,-2.0560856137,1.807463322
H,-2.8683863757,1.5289552647,-0.6357439429
H,-5.3397636454,1.7860572041,-0.7852645793
H,-5.8976874915,-1.7571855476,1.6312849068
H,-6.8049553935,0.1459626404,0.3419964352
C,1.8930879243,1.5975712014,0.1100074911
C,0.6143242967,4.0099257663,-0.6038894267
C,1.404059986,1.7945789058,-1.1934203635
C,1.7339704203,2.6336363023,1.0467438105
C,1.0976093474,3.8274920434,0.6952340969
C,0.773197033,2.9892458165,-1.5476473979
H,1.5316980395,1.0137235604,-1.936956423
H,2.1027073055,2.4986043219,2.0596911994
H,0.9814961668,4.6133497116,1.4361692127
H,0.413165653,3.1258598454,-2.5634648466
H,0.1248578882,4.9392758439,-0.8802103963
H,-2.5120795937,0.2708336955,1.523057

16e

Charge = 1 Multiplicity = 1
C,0.4141810598,-1.1384458942,-1.0393714189
C,-1.9304619443,-0.3217738062,-2.3177397504
C,-0.6631854502,-0.6526013084,-0.2908771389
C,0.3131542086,-1.2307473003,-2.4315369206
C,-0.862130184,-0.8182384398,-3.0641686062
C,-1.8490733527,-0.231709774,-0.9164085706
H,-0.595006221,-0.6132386151,0.7920685447
H,1.1397045351,-1.6167883722,-3.0211163998
H,-0.9379627698,-0.8738690081,-4.1457048251
H,-2.8265502068,0.0171426599,-2.8275959711
C,1.6867293161,-1.6503823879,-0.3213586885
C,2.1146979544,-2.4507931442,2.3719745152
C,1.4257134435,-3.0370813279,0.1591625218

C,2.2392576918,-0.6892154562,0.6990364029
C,2.417571898,-1.1387458519,2.0085826859
C,1.6204646606,-3.4131272757,1.4532717991
H,1.055961618,-3.7411859597,-0.5798197177
H,2.8367573765,-0.4814046586,2.7601756484
H,1.411567367,-4.4231138924,1.7864087895
H,2.2845210048,-2.7516981946,3.4019029794
C,2.5878130807,0.6697875626,0.3095344804
C,3.2650202918,3.3041205853,-0.4232885821
C,2.606998144,1.7105747057,1.2716538119
C,2.9141496447,0.9903003418,-1.0304263992
C,3.2575352914,2.2889949986,-1.3873142838
C,2.9330358535,3.0109527749,0.9048758118
H,2.3226184292,1.5107085406,2.298333312
H,2.9386304179,0.2179108871,-1.7896219346
H,3.5247858773,2.5108300131,-2.4151632236
H,2.9237502674,3.7983475253,1.6512544452
H,3.5286952753,4.3184570931,-0.7058566748
C,-2.9882131126,0.2852561463,-0.1114459707
C,-5.1523976932,1.257598393,1.4168844775
C,-2.7678141153,1.0976408883,1.0157993555
C,-4.3149453451,-0.0287702087,-0.4585384108
C,-5.3857238094,0.4520696586,0.2979195436
C,-3.8386244619,1.5784789802,1.7723547488
H,-1.7543006771,1.3756967034,1.2895269707
H,-4.5107143157,-0.6711791729,-1.3118266512
H,-6.4017035542,0.1904524912,0.0168736757
H,-3.6463101101,2.2114832437,2.6337883236
H,-5.9851608144,1.6318580568,2.0047832514
H,2.4550329404,-1.7564003107,-1.1071937366

16f

Charge = 1 Multiplicity = 1
C,-0.3253713192,-0.3045112145,0.6659449757
C,2.0262570683,0.0854466898,2.1836269377
C,0.9941468535,-0.1261263434,0.0389358801
C,-0.3353654366,-0.3573560058,2.1381431719
C,0.8130980573,-0.1413352516,2.8593251225
C,2.1465822853,0.1001593456,0.7691278138
H,1.0292946917,-0.1522636312,-1.0459320428
H,-1.2864597225,-0.5243823881,2.6336732823
H,0.7974071505,-0.1441616223,3.943256631
H,2.9155087652,0.2725797806,2.7788384121
C,-1.2582272116,-1.3070276319,-0.0281411852
C,-2.9384287383,-3.2386845439,-1.1571277196
C,-0.8350217397,-2.6389436497,-0.1373898454
C,-2.5325960707,-0.9241868342,-0.4941539379
C,-3.362035592,-1.9137224219,-1.0495597464
C,-1.6683257602,-3.6035008603,-0.7037836499
H,0.146518503,-2.9240556887,0.2309663255

H,-4.3448725869,-1.6292457934,-1.4126098544
H,-1.3288789587,-4.6314218642,-0.7831714761
H,-3.5965986156,-3.9814830224,-1.5971282897
C,-3.0275504554,0.4829372679,-0.4076062159
C,-3.9881234286,3.1266624843,-0.252686052
C,-2.4487259492,1.5043017182,-1.1842938934
C,-4.1025608092,0.8057226033,0.4387127964
C,-4.5779042555,2.1167310847,0.5156870966
C,-2.9250187993,2.8169608526,-1.1044467346
H,-1.6466886766,1.2659305767,-1.8775038006
H,-4.5617567646,0.0263404232,1.0399600743
H,-5.40787456,2.3497169481,1.1760643521
H,-2.4719981139,3.5910337601,-1.7165593317
H,-4.359888457,4.1449910904,-0.1922350211
C,3.4615678052,0.3257809761,0.1149567275
C,5.9471449379,0.7410518979,-1.1288793045
C,3.5541981523,1.1062175579,-1.0502886215
C,4.6306086985,-0.2423538843,0.6497529845
C,5.8641437603,-0.0367752889,0.0298965752
C,4.7895841868,1.3118674649,-1.6665308843
H,2.6665057497,1.5761399841,-1.4636364398
H,4.5781574721,-0.8686715763,1.5355448131
H,6.7573114188,-0.4900689524,0.4485166835
H,4.8477236995,1.9246243574,-2.5607519287
H,6.9073618612,0.9016247366,-1.6093033349
H,-0.8005542059,0.702231619,0.4843769746

17

Charge = 0 Multiplicity = 1
C,0.3242703531,0.6762247909,1.5584865412
C,1.6491512618,3.1627843684,1.706539994
C,0.0699016449,1.6673452846,0.5783168164
C,1.2148520727,0.9697625926,2.6061277709
C,1.8765261793,2.1953654902,2.6876354053
C,0.7523804674,2.8951294673,0.6724450721
H,1.40453896,0.2070602497,3.3563093282
H,2.5641615073,2.3883823122,3.5058426229
H,0.5498337401,3.6628177276,-0.0686114086
H,2.1528746509,4.1239500364,1.7518374997
C,-0.3242703531,-0.6762247909,1.5584865412
C,-1.6491512618,-3.1627843684,1.706539994
C,-1.2148520727,-0.9697625926,2.6061277709
C,-0.0699016449,-1.6673452846,0.5783168164
C,-0.7523804674,-2.8951294673,0.6724450721
C,-1.8765261793,-2.1953654902,2.6876354053
H,-1.40453896,-0.2070602497,3.3563093282
H,-0.5498337401,-3.6628177276,-0.0686114086
H,-2.5641615073,-2.3883823122,3.5058426229
H,-2.1528746509,-4.1239500364,1.7518374997
C,0.9090683499,-1.4958666613,-0.5357370718

C,2.772029069,-1.3067013614,-2.6510628091
C,0.5452102725,-1.8301973734,-1.8525586246
C,2.2277918859,-1.068161896,-0.3000013229
C,3.1493222684,-0.9749421263,-1.3458721866
C,1.4648897274,-1.7359864279,-2.8996878667
H,-0.4715623121,-2.1509253382,-2.0599083377
H,2.5398906867,-0.8215535987,0.7092950439
H,4.1649269225,-0.6497799302,-1.1381765661
H,1.1576240228,-1.9929594028,-3.9095652418
H,3.4888571892,-1.2336202565,-3.4638342184
C,-0.9090683499,1.4958666613,-0.5357370718
C,-2.772029069,1.3067013614,-2.6510628091
C,-2.2277918859,1.068161896,-0.3000013229
C,-0.5452102725,1.8301973734,-1.8525586246
C,-1.4648897274,1.7359864279,-2.8996878667
C,-3.1493222684,0.9749421263,-1.3458721866
H,-2.5398906867,0.8215535987,0.7092950439
H,0.4715623121,2.1509253382,-2.0599083377
H,-1.1576240228,1.9929594028,-3.9095652418
H,-4.1649269225,0.6497799302,-1.1381765661
H,-3.4888571892,1.2336202565,-3.4638342184

17a

Charge = 1 Multiplicity = 1
C,-0.2000551047,-0.7123984175,-0.942607343
C,-0.7054077502,-3.4661916422,-1.245128852
C,0.7017804881,-1.5491406145,-1.6272399176
C,-1.3892018104,-1.2696119455,-0.4044525713
C,-1.6116165502,-2.6487711587,-0.5692829255
C,0.4592740694,-2.9126131247,-1.7809555869
H,1.5897663352,-1.1120932932,-2.074209336
H,-2.5064005333,-3.0873864333,-0.1385188534
H,1.1682171667,-3.5321125305,-2.3213592683
H,-0.9092268735,-4.5274396584,-1.3500128924
C,0.0996735334,0.7492158542,-0.9052240764
C,0.6871265095,3.5079111881,-1.1858013226
C,-0.818711944,1.635518988,-1.4848477285
C,1.3491518004,1.2712417517,-0.448821605
C,1.6243528151,2.65200837,-0.6268032867
C,-0.5412316016,2.9950678814,-1.6187396206
H,-1.7562675672,1.2424439388,-1.8624534647
H,2.5599845086,3.0625223565,-0.2645051202
H,-1.2759203343,3.6507240642,-2.0755046178
H,0.9084241678,4.5654382866,-1.2827336548
C,2.3513453453,0.475188989,0.2573240421
C,4.3673150536,-0.9733252478,1.7205985852
C,3.7437658466,0.7611484984,0.062360646
C,1.9806343037,-0.5444627148,1.1938895233
C,2.9255372064,-1.2207700743,1.9046747021
C,4.7052632681,0.0710628981,0.736812173

H,4.0315947394,1.5054604278,-0.6699678566
H,0.9332689975,-0.7520011865,1.3648572767
H,2.6255993281,-1.9605852253,2.6405317366
H,5.7552773696,0.2745892731,0.5496761701
C,-2.4101548675,-0.4769880848,0.3444359836
C,-4.3911997327,0.9347967929,1.7781150484
C,-3.7699502355,-0.5934535032,0.0035540345
C,-2.0646499651,0.3581727215,1.4211586052
C,-3.0438667854,1.0551403526,2.1320307192
C,-4.7503296389,0.107681888,0.7098867175
H,-4.0593974562,-1.2246114623,-0.8316546055
H,-1.0260658007,0.4588896991,1.7188637021
H,-2.7533956016,1.6880139138,2.9655292477
H,-5.7930613345,0.0091384242,0.4225258885
H,-5.1522433041,1.4785658094,2.3297357545
H,4.8327467201,-0.730493874,2.6937001456
H,4.8813481789,-1.9161898759,1.4577270238

17b

Charge = 1 Multiplicity = 1
C,-0.1599115198,-0.7971339027,-0.9188317249
C,-0.6793549375,-3.5481312193,-1.1592706319
C,0.7264073893,-1.6423967298,-1.6074036277
C,-1.3373551664,-1.3360056603,-0.3458418309
C,-1.5699857116,-2.717665967,-0.481074745
C,0.4754544465,-3.0072169785,-1.7304181157
H,1.6153805348,-1.219590643,-2.065748849
H,-2.4603721103,-3.1432773557,-0.0288464127
H,1.1727969355,-3.6396240399,-2.2707670763
H,-0.8859536243,-4.6109601666,-1.2395804918
C,0.1231316032,0.6748543296,-0.9201071248
C,0.6723241031,3.4840016756,-1.1616648175
C,-0.7833297802,1.5058837658,-1.5322525755
C,1.3453978172,1.2555595723,-0.4113382035
C,1.5847191079,2.6606738273,-0.5773263123
C,-0.5955495439,2.9553490793,-1.6821401805
H,-1.6875849733,1.0799717192,-1.9556210893
H,2.4944798131,3.0807372356,-0.1667265772
H,0.8600376314,4.5505310144,-1.2349624491
C,2.3736364304,0.4828253519,0.2907962005
C,4.3879043017,-0.9016479709,1.6872015972
C,3.7393907627,0.8073351202,0.1056511319
C,2.0446865522,-0.55405111,1.1947672706
C,3.0415457935,-1.2198240824,1.8999050636
C,4.7337159946,0.106184083,0.7799542523
H,4.0242766913,1.5772389716,-0.6030386124
H,1.010129031,-0.817874257,1.3710516889
H,2.76929532,-1.9927671117,2.6109855264
H,5.7766083604,0.3472271364,0.6025123247
H,5.1635839678,-1.4376771261,2.2251308327

C,-2.34792988,-0.516322478,0.3891968876
C,-4.3105296579,0.9499828984,1.7932904608
C,-3.7076974944,-0.6109459692,0.0406787086
C,-1.9931563545,0.3249023073,1.4588857249
C,-2.9636135572,1.049025899,2.1549963271
C,-4.6788755321,0.1164766724,0.7329999984
H,-4.0038665756,-1.2493277079,-0.7866561011
H,-0.9556853279,0.4024013079,1.767659863
H,-2.6667245444,1.6837786215,2.9847501359
H,-5.7218070934,0.0323631978,0.4419915326
H,-5.0647107891,1.5135746396,2.3342432804
H,-1.448324309,3.4903506191,-1.2234187718
H,-0.7120066246,3.231843111,-2.7466024073

17c

Charge = 1 Multiplicity = 1
C,0.3923652015,1.3043147442,-0.4824591374
C,1.6776464896,3.4942533608,0.7459905841
C,1.6424488317,1.114307834,0.1862942891
C,-0.1900752223,2.5963102644,-0.4767357037
C,0.4422201934,3.6789896148,0.1193924224
C,2.2574359586,2.2257005651,0.7792713283
H,-1.1164148821,2.7727293607,-1.0097650152
H,-0.0171560333,4.6610026312,0.0813760555
H,3.1943913492,2.0803405736,1.3070595661
H,2.1838468813,4.3309257161,1.2170750058
C,-0.2684699858,0.255461181,-1.260844133
C,-1.6048888,-1.6296931191,-2.9988577146
C,0.4551026598,-0.6390025855,-2.0499798685
C,-1.7759371941,0.183141216,-1.2886213646
C,-2.368362946,-0.7978532915,-2.2381594735
C,-0.191435874,-1.5403848993,-2.895948342
H,1.5366446812,-0.6037197667,-2.0557227136
H,-3.4523819678,-0.8217150478,-2.2929621058
H,0.418569689,-2.1843705133,-3.5230144098
H,-2.0534185794,-2.3407847755,-3.6829827921
C,-2.3947035905,-0.0227247814,0.1173150541
C,-3.5428840512,-0.4167522519,2.6441690385
C,-2.22955634,-1.2448335551,0.7838739086
C,-3.1507843219,0.9928834699,0.7127208768
C,-3.7203733779,0.796121766,1.9744258175
C,-2.7984076525,-1.4374593618,2.0448954895
H,-1.6657160815,-2.0505563297,0.3231961923
H,-3.3010589944,1.9375019361,0.1992202147
H,-4.3029227897,1.5919648239,2.4281154304
H,-2.6623240524,-2.3863540923,2.5545588447
H,-3.9855049809,-0.5685189312,3.6237005687
C,2.2937811195,-0.2134229626,0.3702193389
C,3.5747455522,-2.6845756955,0.7949912733
C,3.6401775923,-0.3943145838,0.0065192434

C,1.6053136166,-1.2840832481,0.9668633822
C,2.2412874642,-2.5089331735,1.1776051725
C,4.2724546056,-1.6226397513,0.2102889059
H,4.1861672643,0.423244122,-0.4550918561
H,0.5775905545,-1.1493948025,1.2887622479
H,1.6978941456,-3.3223266221,1.6492050502
H,5.3087782329,-1.749298072,-0.0881365756
H,4.0684170091,-3.6377406463,0.958218671
H,-2.1462280641,1.1656864298,-1.6257200067

17d

Charge = 1 Multiplicity = 1
C,0.1647519966,1.0419500116,0.820159809
C,0.9390537287,0.8661508535,3.5095806345
C,-0.6337319422,1.591572071,1.8339096528
C,1.3858876461,0.4099316127,1.1455521566
C,1.7420004835,0.3278791049,2.50586012
C,-0.2559094508,1.5059036066,3.1724307795
H,-1.5690766106,2.0803659724,1.5765968502
H,2.6776178587,-0.1559992973,2.7685259777
H,-0.891436671,1.9324654324,3.9419870369
H,1.2482524031,0.791018551,4.5474723336
C,-0.3415397118,1.1678221649,-0.6400177548
C,-2.4111904305,2.7507040902,-1.7959186197
C,-0.1304590629,2.5678311632,-1.1023885815
C,-1.7294798567,0.6155514973,-0.8463229437
C,-2.7016622405,1.4427018027,-1.4129089571
C,-1.1231518883,3.3256358473,-1.6454396272
H,0.8664938485,2.976600208,-0.9774179773
H,-3.6976586906,1.0614701997,-1.6002567518
H,-0.9433183578,4.3460186753,-1.9635185313
H,-3.2016995871,3.3457625869,-2.2440356659
C,-2.0424940318,-0.7591413256,-0.4793439064
C,-2.6742404724,-3.4160199414,0.209786422
C,-3.371364301,-1.1297600821,-0.1544786704
C,-1.0408169572,-1.7572273232,-0.4445521335
C,-1.3572917612,-3.0702831506,-0.1146001939
C,-3.6777158769,-2.4398209643,0.1946257822
H,-4.1571566202,-0.3835111065,-0.1316875035
H,-0.0193909739,-1.5193129176,-0.7152879368
H,-0.579130877,-3.8264166447,-0.1154684046
H,-4.6969614237,-2.7010775041,0.4593900204
H,-2.9175384883,-4.4407342322,0.4724744458
C,2.3061302529,-0.2035769245,0.1410521032
C,4.0961254474,-1.4006968068,-1.683519469
C,2.898136393,0.5498278275,-0.8886643045
C,2.6421647005,-1.5665010263,0.2475984986
C,3.5252829416,-2.1596712323,-0.6566405203
C,3.7828496928,-0.0437113252,-1.7938300252
H,2.7013515622,1.6144737155,-0.9647198648

H,2.2007867296,-2.1638006175,1.0400800676
H,3.7663243928,-3.2140186637,-0.5591169761
H,4.2346055643,0.5593139167,-2.5756907072
H,4.7840605529,-1.8612794963,-2.3859537461
H,0.3422220692,0.5518342308,-1.2547648883

TS7

Charge = 1 Multiplicity = 1
C,3.8805681369,1.6295713172,0.4393422206
C,1.1063922308,0.936616183,0.2985387464
C,3.3150986269,0.6622383433,1.3492918907
C,3.0329092547,2.2134977958,-0.5643929242
C,1.7108005791,1.8795871157,-0.6217859804
C,1.9011200699,0.3613311882,1.260611675
H,3.4554558883,2.9396765411,-1.2493306307
H,1.0837808627,2.3398480499,-1.3779664827
H,1.4809958145,-0.2998863348,2.0101019286
C,4.3586822932,-0.1074094978,0.2249343091
C,5.9114560767,-2.0846644545,-0.9725542501
C,5.6519946906,-0.3773847864,0.716015606
C,3.8486162907,-0.8212808827,-0.8754773497
C,4.6272512409,-1.8075694318,-1.4649231781
C,6.4207887687,-1.3702180555,0.1163307778
H,6.045142954,0.1799801298,1.5604844966
H,2.8589802115,-0.610226567,-1.2633826482
H,4.2356337785,-2.362608797,-2.3105835769
H,7.413649248,-1.5826669407,0.4977840109
H,6.5130406051,-2.8564733502,-1.4415981465
C,-0.341400598,0.6408428895,0.2069337334
C,-3.1242967203,0.0734298255,0.0581514546
C,-0.8343867984,-0.645557948,0.4913392535
C,-1.2646189351,1.6370946156,-0.1590675506
C,-2.6274230193,1.3593582586,-0.2242157684
C,-2.1965906106,-0.9227233483,0.415306764
H,-0.1451826113,-1.4471456423,0.7385914621
H,-0.9265324394,2.6493869066,-0.3579960818
H,-3.316302065,2.1603014243,-0.4728096183
H,-2.5397570327,-1.9345613962,0.6060032958
C,-4.5777722749,-0.2231629312,-0.0214552429
C,-7.3396438014,-0.7855949611,-0.1695546774
C,-5.186195936,-1.0993807592,0.8965110384
C,-5.3817347066,0.364849446,-1.01559208
C,-6.7479440435,0.0860579278,-1.0892402428
C,-6.5526522822,-1.3768850434,0.8237311828
H,-4.5921147427,-1.5486833093,1.6867239312
H,-4.9324170303,1.0264530525,-1.7501599353
H,-7.3480809517,0.5440526986,-1.8700950234
H,-7.0034013606,-2.0490306139,1.5480972824
H,-8.4023009118,-1.0018045654,-0.2264021785
H,4.8260738651,2.106009191,0.6651273829

H,3.7887504351,0.4962199567,2.3100600039

Imaginary Mode = -256.9184

TS8

Charge = 1 Multiplicity = 1
H,3.2808802106,-1.7485659407,0.8220607831
C,3.6057650024,-1.1803097491,-0.0415865491
C,4.445041553,0.2847602108,-2.2887016835
C,3.5840510476,0.2287385187,-0.0243996669
C,4.0529468617,-1.8411537575,-1.1764623177
C,4.4726768845,-1.113448475,-2.3003325185
C,3.9988712922,0.9607390311,-1.1577918835
H,4.0744075545,-2.9254999066,-1.189417141
H,4.8188758469,-1.6397710169,-3.1839697192
H,3.9798391062,2.0459406224,-1.1472077961
H,4.7701087105,0.8483446816,-3.1564425441
C,3.6283657894,1.100643236,1.4402756364
C,1.6361853988,-0.4599405776,2.6856531505
C,3.9310698184,0.3609987995,2.650117769
C,2.2829285397,1.0561382914,0.9149180929
C,1.2696334798,0.2342476002,1.5500764305
C,2.9542395346,-0.3885015934,3.2385121136
H,4.9249617258,0.4409792531,3.0750571829
H,3.1623464986,-0.9342732962,4.1529590819
H,0.9050447353,-1.0807144709,3.1921704479
C,-0.0970281154,0.2091010588,0.9944772899
C,-2.7280276236,0.1250197311,-0.0888270761
C,-1.2184356472,0.0172148881,1.8245364885
C,-0.3196053859,0.3686513132,-0.3862847843
C,-1.6072379266,0.3236622978,-0.9149410714
C,-2.5030091246,-0.0245170506,1.2932067979
H,-1.0915560728,-0.0816966599,2.8979214328
H,0.5168658656,0.5106786872,-1.0636020101
H,-1.742363843,0.46136725,-1.9829663948
C,-4.1009155484,0.0774956316,-0.6532840557
C,-6.7104110628,-0.0206968966,-1.7251338455
C,-5.1899330367,0.6305257916,0.046061271
C,-4.3467874541,-0.5253605796,-1.9010436231
C,-5.6376582767,-0.5748277195,-2.4306878276
C,-6.4804113326,0.5828245468,-0.4847872149
H,-5.0244096242,1.121871131,1.000144571
H,-3.5275119134,-0.9799170785,-2.4500040615
H,-5.8059803584,-1.0538467896,-3.3908284292
H,-7.3048362237,1.0237757791,0.0679691273
H,-7.7143965571,-0.058880385,-2.1371177776
H,4.2446312976,1.9663899019,1.2250381724
H,1.9536924421,1.8393297176,0.2436672027
H,-3.3429142384,-0.1875160371,1.9609970994

Imaginary Mode = -215.2357

TS9

Charge = 1 Multiplicity = 1
C,3.722175312,1.2853370069,0.6305445628
C,0.8927719591,1.5034830367,0.25633588
C,2.8120578929,0.4642945063,1.3921966873
C,3.1825101649,2.1921573274,-0.3474948725
C,1.8320418353,2.2912116326,-0.517190205
C,1.3872478106,0.624555352,1.1886337796
H,3.864775719,2.8105356898,-0.919543103
H,1.4418776425,2.9895452452,-1.2497139577
H,0.7220832378,0.0625465953,1.8342389474
C,3.6774333194,-0.4793871795,0.2462515985
C,4.6682619363,-2.7239301355,-1.0701932485
C,4.7791890578,-1.1774240333,0.7807676391
C,3.0788057197,-0.9011911942,-0.9558800322
C,3.5765933354,-2.0228270026,-1.6043915003
C,5.2668896999,-2.3008508829,0.1207999086
H,5.242025765,-0.8448089218,1.7045616364
H,2.2378501128,-0.3615704306,-1.3753349772
H,3.1157883085,-2.35350891,-2.5290179749
H,6.111227891,-2.8414095826,0.5345835411
H,5.0510947076,-3.5983071022,-1.5864432743
C,-0.5656128045,1.6871038377,0.0575351474
C,-3.3285978362,2.0193959404,-0.2985208767
C,-1.4379488867,0.5894379471,0.1455252445
C,-1.0969952556,2.9588257789,-0.2210922642
C,-2.4723176861,3.1175657342,-0.3905286518
C,-2.8229135559,0.7341836622,-0.0321071262
H,-1.0287852579,-0.4025289126,0.3085813691
H,-0.4475128309,3.8266061815,-0.276993827
H,-2.8805166582,4.1048366391,-0.5836264333
H,4.7419121015,1.4275687707,0.964947264
H,3.1258259559,0.0715724465,2.3526818236
H,-4.3978397315,2.1630838384,-0.4177620258
C,-3.7275910807,-0.4442322075,0.0466357898
C,-5.446272065,-2.6786619339,0.1916658573
C,-4.8178504074,-0.574269215,-0.8329909679
C,-3.5160122818,-1.4565649558,1.0003261382
C,-4.3663065615,-2.5621817536,1.0722634024
C,-5.6682969525,-1.6796182917,-0.7612206673
H,-4.9902786833,0.182788311,-1.5922475103
H,-2.6956696628,-1.3680449764,1.706564039
H,-4.1895658981,-3.3277616756,1.8222333703
H,-6.4997386909,-1.7632136453,-1.455069657
H,-6.1078174773,-3.5380151075,0.247767357

Imaginary Mode = -250.480

TS10

Charge = 1 Multiplicity = 1
H,-2.7155744547,0.7983684194,-0.4150716092
C,-3.1571433483,-0.0267140741,-0.9613422969
C,-4.3100831946,-2.1592000675,-2.3833451739
C,-2.6436610624,-1.3313301404,-0.8345208711
C,-4.238711146,0.1993116608,-1.8008475927
C,-4.8175320411,-0.8626750615,-2.5119919823
C,-3.2259323424,-2.4006056204,-1.5449764993
H,-4.6338183817,1.2042602214,-1.903593957
H,-5.6638868333,-0.6757136731,-3.1649787434
H,-2.8315125916,-3.4069171056,-1.4455005316
H,-4.7558304736,-2.9814981313,-2.932393929
C,-1.9172463755,-1.8452136983,0.723295241
C,-0.1895505549,0.433933798,0.7620467203
C,-1.9661176809,-0.9091244746,1.8168471259
C,-0.9995519261,-1.5954392594,-0.3611660977
C,-0.1353802239,-0.4369126412,-0.2971588527
C,-1.1385098417,0.176132811,1.8258700843
H,-2.6359940909,-1.108917665,2.6453836506
H,-1.1528672981,0.8487249927,2.6776521687
C,2.8493716722,0.3480303853,0.4077847263
C,4.3876997435,-1.9892959325,0.0615368209
C,2.813176195,-0.6838032088,1.3618826474
C,3.6768253525,0.192617012,-0.7173771476
C,4.4366369641,-0.9676728439,-0.8913345668
C,3.5757716406,-1.8410380967,1.1911400368
H,2.1979879837,-0.572609192,2.2501194508
H,3.7155774083,0.9791770675,-1.465675462
H,5.0648935376,-1.0718419012,-1.7712009842
H,3.5420389097,-2.6230425613,1.9442173675
H,4.9806765881,-2.889366712,-0.070901555
H,-2.3313689122,-2.8345834767,0.8716193101
H,-0.6746253323,-2.4158155659,-0.9904536321
H,0.5710735923,-0.2894545705,-1.1052871227
C,0.6783996049,1.641490443,0.841378616
C,2.1441382728,4.0436586618,0.8544430111
C,0.0530631593,2.8733180429,1.108745794
C,2.0765526646,1.6103598574,0.6071999461
C,2.7811175147,2.8263493554,0.6117539084
C,0.772080516,4.0675710544,1.1125832193
H,-1.0190582782,2.8990535255,1.2814954244
H,3.854227715,2.8064261448,0.4475586028
H,0.2627973809,5.0058314032,1.3088446165
H,2.719848859,4.9641709075,0.8557201979

Imaginary Mode = -258.5493

TS11

Charge = 1 Multiplicity = 1
H,3.0102857634,-1.6351111709,0.9126143331
C,3.6238332708,-1.0750877966,0.2167120239
C,5.2156927144,0.3692283611,-1.5956529672
C,3.5926043681,0.3337937467,0.2027335731
C,4.4545910321,-1.7460695311,-0.6686677612
C,5.2510287112,-1.0288444546,-1.5744155348
C,4.3869292117,1.0552614029,-0.7141878652
H,4.4838067886,-2.8302259,-0.6564957822
H,5.8968845461,-1.5632324427,-2.2635440346
H,4.3577783761,2.140300496,-0.7280529156
H,5.8306975613,0.9241791389,-2.2956616553
C,3.1067439481,1.2246486296,1.5742815683
C,0.7964237573,-0.3290619052,2.0270064662
C,2.9475683728,0.5001629776,2.8200010779
C,2.0455512923,1.1674865264,0.5947684891
C,0.8695232943,0.3501150254,0.8289715447
C,1.8235043582,-0.2463559539,3.0214134809
H,3.7182577884,0.5889971915,3.5769758963
H,1.6842653099,-0.7810308693,3.9552914295
H,-0.0699248142,-0.9460313076,2.2400519617
C,-0.2004671567,0.3130012461,-0.1909950122
C,-2.2296547688,0.1945679387,-2.1284751465
C,-1.544407886,0.1383433798,0.1841259682
C,0.1113695485,0.4364191425,-1.5574102533
C,-0.901261278,0.3721557444,-2.5148014962
C,-2.5716740002,0.076638369,-0.7682167184
H,-1.7990578648,0.0551613874,1.2356112375
H,1.1405606795,0.5551989937,-1.8799252986
H,-0.6528462802,0.4651831658,-3.5675130652
H,-3.0094355722,0.1725001823,-2.8832353349
C,-3.9887017011,-0.1004395781,-0.3500321593
C,-6.6779095154,-0.4408895784,0.4341976705
C,-4.4971487313,0.5567675395,0.7847655419
C,-4.8541618406,-0.9313126834,-1.0844553458
C,-6.1849289915,-1.1002268856,-0.6961306792
C,-5.8282749896,0.3885770546,1.172795333
H,-3.8552590513,1.2225454698,1.3541821951
H,-4.4790407855,-1.4654518146,-1.9524717478
H,-6.8339611836,-1.7524681112,-1.2732187132
H,-6.2030151948,0.9130351141,2.0470009738
H,-7.7130084327,-0.5712802227,0.7355170867
H,3.7624449951,2.0881022699,1.585370606
H,1.9837939798,1.940749712,-0.1608289707

Imaginary Mode = -203.3688

TS12

Charge = 1 Multiplicity = 1
C,1.1593646538,1.7564213363,0.2367333572

C,1.119318652,0.5451166149,2.7727747755
C,2.3466374306,1.7573081803,1.0610542505
C,-0.0702911687,1.1396566959,0.7029937036
C,-0.0509347916,0.5477684818,1.9464405938
C,2.2801214641,1.1393632049,2.3675918376
H,-0.9620528181,0.1173072714,2.3464606327
H,3.1522968887,1.1810361188,3.0098957179
C,2.4995160121,0.6985276086,-0.2896621168
C,3.7489595513,-1.0018610957,-2.1122772056
C,3.1985402455,1.2365309047,-1.3918783773
C,2.4296399604,-0.6964619514,-0.1055108048
C,3.0543303801,-1.5359705667,-1.016918338
C,3.8206201781,0.38276136,-2.2964301052
H,3.2540208459,2.3111220844,-1.5344346775
H,1.906282786,-1.1162006323,0.7448679035
H,3.0052495466,-2.6100038503,-0.8728812394
H,4.3588455888,0.797789001,-3.141616635
H,4.2352018919,-1.6671626828,-2.8183422688
H,1.0877376961,2.459878551,-0.5826678878
H,3.1047578085,2.5152512003,0.9010331261
H,1.0540497511,0.0977738012,3.759286744
C,-1.3128894976,1.2981358341,-0.0991420275
C,-3.726003723,1.8150857491,-1.4619429074
C,-2.2459641865,0.2488154449,-0.3033055766
C,-1.6071837719,2.5796084359,-0.605955748
C,-2.7975162387,2.8421364532,-1.2802730242
C,-3.4444216184,0.5377246307,-0.9776654644
H,-0.9154111779,3.3976506651,-0.4294748429
H,-3.0013322532,3.8431541205,-1.6470495734
H,-4.1537343007,-0.2673459863,-1.143771267
H,-4.6593225534,2.0029798169,-1.9836907317
C,-2.005314068,-1.1625411847,0.1209423781
C,-1.6154734824,-3.8577379791,0.8487967704
C,-2.9079242013,-1.8112315252,0.9808516892
C,-0.913073523,-1.8907460713,-0.379718213
C,-0.7189198023,-3.2263509719,-0.018948394
C,-2.7119085768,-3.1460730255,1.3454954167
H,-3.7596985649,-1.2636609941,1.3743619607
H,-0.2270517947,-1.4159364641,-1.0744767573
H,0.1272005046,-3.7744152913,-0.4235504193
H,-3.4161136894,-3.6285743926,2.0169534699
H,-1.4657060335,-4.8961717505,1.1286906454

Imaginary Mode = -227.2265

TS13

Charge = 1 Multiplicity = 1
C,0.9699422483,-1.1477823173,0.1587739732
C,0.6740979231,-0.1682905833,2.793642244
C,1.3617490087,-1.9345390546,1.2661214514

C,0.4395442342,0.1414306658,0.3841266194
C,0.2949008108,0.6123368361,1.6746808847
C,1.2108392601,-1.4512636806,2.5549259712
H,1.7623862529,-2.9315083546,1.1117918469
H,0.1493665727,0.7719771922,-0.4482724839
H,-0.0953003312,1.6128748575,1.8230488329
H,1.4865672623,-2.0881464391,3.3876457724
C,1.9915776127,-1.4257338059,-1.2853138094
C,-0.0745622393,-0.4144704178,-2.9954756076
C,2.2450666487,-0.3098658247,-2.1612294887
C,0.6628476212,-1.9999681907,-1.2571483463
C,-0.3457706365,-1.4657554596,-2.1584538323
C,1.2576202884,0.1625285384,-2.9741916549
H,3.2458578297,0.1050746039,-2.2002309082
H,-1.3239551207,-1.9330706112,-2.1468048837
H,1.4855152555,0.9643717543,-3.6686954645
H,2.8364832081,-1.9824228122,-0.8996229858
H,0.5381642045,-3.0331165961,-0.9508363092
C,-1.0885377621,0.1174452491,-3.9397533094
C,-3.0300338957,1.135809796,-5.7074558609
C,-1.1591199164,1.495846778,-4.2121739728
C,-2.0031186005,-0.7430197818,-4.5746377711
C,-2.9637224289,-0.2376967987,-5.4511384111
C,-2.1255667648,2.0003272644,-5.0843570876
H,-0.4750624992,2.1835787179,-3.7242589242
H,-1.9471670146,-1.813652828,-4.4036408295
H,-3.6541152596,-0.9177485405,-5.9408331054
H,-2.1721023591,3.0683618379,-5.2739612336
H,-3.7774884321,1.5278485825,-6.3904339337
C,0.5120334669,0.3469840626,4.1691189403
C,0.1989368889,1.3261151243,6.7925035753
C,1.424004002,-0.0060628766,5.1836110885
C,-0.5599109529,1.2008303849,4.496319092
C,-0.7168950126,1.6803079274,5.7965644683
C,1.2707821538,0.4833930326,6.4804465505
H,2.2735808854,-0.6409062634,4.9530286961
H,-1.2922999463,1.4665846859,3.7408485579
H,-1.5570797345,2.3260158127,6.0328275527
H,1.9914673889,0.2114695022,7.2455313648
H,0.0784779394,1.7035081304,7.8033628114

Imaginary Mode = -202.4403

TS14

Charge = 1 Multiplicity = 1
C,1.4916444586,0.2208738813,-0.4403030214
C,0.6519041312,0.6022993256,2.2344277947
C,1.9337094755,-0.6486550645,0.5853095062
C,0.6117045549,1.2771029648,-0.1109443389
C,0.2087667695,1.4590664198,1.1971584318

C,1.523563055,-0.4542605408,1.8928804039
H,2.5975439782,-1.4747550241,0.3494226015
H,0.2621300412,1.9610216533,-0.8756567294
H,-0.4390859427,2.2963015366,1.4301513623
H,1.8632189096,-1.1459396564,2.6551530146
C,2.4186120691,0.2413565269,-1.8185708962
C,0.2969884586,0.9824762528,-3.5365356644
C,2.4819908423,1.4829687157,-2.5778463259
C,1.2689921744,-0.6238091439,-2.0103018614
C,0.1764509844,-0.2211318896,-2.8762991892
C,1.4490913653,1.8256933942,-3.3967611865
H,3.369937771,2.0988165918,-2.490490955
H,1.4929395151,2.7422616924,-3.9759450691
H,-0.4969339959,1.3108895789,-4.1989784124
H,3.3675822617,-0.2114478862,-1.5506121332
H,1.3503317251,-1.6716264482,-1.7498681831
C,0.2142822397,0.8089381098,3.630060649
C,-0.6209871384,1.2023603207,6.2926074196
C,1.0613041618,0.4803970603,4.7075681028
C,-1.0599121678,1.3393260277,3.9152088902
C,-1.4740616823,1.5282354391,5.2332366117
C,0.6486022676,0.6802800277,6.0244202191
H,2.0582719685,0.0958538389,4.5175486524
H,-1.7430136801,1.5775684306,3.1063091677
H,-2.4645841427,1.9253026788,5.4328638095
H,1.320581682,0.4340649515,6.8407860567
H,-0.9426488056,1.3543024952,7.3183722163
C,-0.9762090973,-1.1295444306,-3.0566587671
C,-3.1853920109,-2.8469029542,-3.3863965804
C,-1.6569599685,-1.1976691821,-4.2876960731
C,-1.4180007137,-1.9450879078,-1.9976379306
C,-2.514950482,-2.7928447437,-2.1612953221
C,-2.7510882825,-2.046761646,-4.4489787083
H,-1.313534878,-0.60615228,-5.1305705488
H,-0.9231841976,-1.9015881198,-1.0323959266
H,-2.8469240375,-3.4067844989,-1.3296434544
H,-3.2571917096,-2.0928875066,-5.4084065686
H,-4.0354817573,-3.5099091008,-3.5146910739

Imaginary Mode = -155.3100

TS15

Charge = 1 Multiplicity = 1
C,1.8397229504,-1.4382621645,0.6342223514
C,1.5858646905,-0.9527418438,3.3545072751
C,2.5516453908,-2.2368192705,1.5492119541
C,1.0109804515,-0.3957862702,1.0810765057
C,0.8737062015,-0.13847894,2.4471149361
C,2.4098514843,-1.9878540495,2.9106926045
H,3.1976025456,-3.0373901654,1.2038450765

H,0.4568476361,0.2019746907,0.3673491681
H,2.9529840465,-2.5952972179,3.6265657333
H,1.5076700074,-0.757604325,4.4189642758
C,2.6945176954,-1.2604675107,-0.9546846656
C,0.2353709968,-0.6473766311,-2.284982793
C,2.5354017139,-0.0161305943,-1.6580983165
C,1.5804853607,-2.1757489319,-0.9035198158
C,0.362978218,-1.8369939566,-1.6097439767
C,1.360358438,0.2662378958,-2.2924083045
H,3.3713133024,0.6729538399,-1.6980678681
H,-0.4290868284,-2.5769849057,-1.6287495654
H,1.2629669359,1.2035831027,-2.8295412325
H,3.6952010475,-1.601132851,-0.7202063434
H,1.7631871873,-3.2294110658,-0.725612311
C,-0.0041748168,0.9611218795,2.9286967865
C,-1.6728137786,3.0379950585,3.8425464821
C,-0.8041993328,0.7880780316,4.0720615213
C,-0.0522129947,2.192860653,2.2524416531
C,-0.8796306018,3.2220725571,2.7058649569
C,-1.6320514412,1.8176277664,4.5237693523
H,-0.7981533221,-0.1620287539,4.5978560776
H,0.5776484147,2.3582320132,1.3832849868
H,-0.8972413315,4.1698219129,2.176159834
H,-2.2495973549,1.663073637,5.4034467541
H,-2.3159957195,3.8386893062,4.1948552713
C,-1.0022140421,-0.303913681,-3.0255850925
C,-3.3524066084,0.3148815308,-4.4449920898
C,-0.9393255083,0.3882414586,-4.2491083701
C,-2.2627426148,-0.674343627,-2.5219642336
C,-3.4273985232,-0.365669946,-3.2256746784
C,-2.1051190096,0.6891437242,-4.9546440594
H,0.0221298776,0.6684704731,-4.6685886074
H,-2.3342730375,-1.1816548325,-1.5648270114
H,-4.3926008269,-0.6493843461,-2.8174562491
H,-2.0382216188,1.2118529566,-5.9038442036
H,-4.2589750706,0.5545633228,-4.9922337292

Imaginary Mode = -256.6475

TS16

Charge = 1 Multiplicity = 1
C,0.2415299612,-0.1424707024,1.3545872487
C,-0.5164788963,0.1039922416,4.025392815
C,0.5211773959,-1.2109587227,2.2519221789
C,-0.4007506662,1.0287209936,1.80379775
C,-0.7796884914,1.1500101588,3.1301108713
C,0.1238093905,-1.0550012028,3.5863862424
H,-0.6033411081,1.8380093273,1.112593278
H,-1.2771187898,2.0527153045,3.46753081
H,0.324390368,-1.8613050666,4.2838783693

H,-0.809950196,0.1935155805,5.0663856198
C,1.439284562,0.0303177528,0.0155409691
C,-0.4281995764,1.4554074881,-1.6185236102
C,1.7457258283,1.3552960299,-0.458209648
C,0.1650510653,-0.5486201477,-0.3215337424
C,-0.7501579765,0.203300549,-1.154512384
C,0.8539859319,2.0222041003,-1.2459820213
H,2.715196619,1.7820954503,-0.2275604297
H,-1.6959327982,-0.2613203557,-1.4088662723
H,1.1277456079,2.9917115607,-1.6486153975
H,2.2518365679,-0.6064424451,0.3434879066
H,0.0581373176,-1.6273537588,-0.3215811629
C,1.2107619808,-2.475877872,1.8514094334
C,2.502336776,-4.8800868726,1.1871915874
C,0.4758257856,-3.5613648578,1.3469339023
C,2.5967513786,-2.6116000405,2.0335228716
C,3.237906182,-3.8077229756,1.6997348526
C,1.1204719143,-4.756231308,1.0161289096
H,-0.6009399771,-3.4780403694,1.2293142335
H,3.1703242854,-1.7866479371,2.4472899778
H,4.3095488185,-3.9011366104,1.8466030815
H,0.5406283581,-5.5896428711,0.6315205272
H,3.0014987385,-5.8093559913,0.9304685315
C,-1.3367795526,2.2205064193,-2.5069164366
C,-3.0824846986,3.6760919535,-4.1688625728
C,-1.4369152518,3.6194530775,-2.3930990159
C,-2.121663842,1.5642031439,-3.473038997
C,-2.9849632774,2.2865445311,-4.2969650996
C,-2.3069701419,4.3393470516,-3.2136534487
H,-0.8557918051,4.1490178067,-1.6442034712
H,-2.0367953945,0.4894552198,-3.6000883806
H,-3.5743283395,1.7652029303,-5.0450533118
H,-2.3800560953,5.4169510581,-3.1035468062
H,-3.7546171487,4.2371103086,-4.8109195787

Imaginary Mode = -254.1726

TS17

Charge = 1 Multiplicity = 1
C,1.3894311939,-0.1408557301,1.3773301844
C,0.4657530754,-0.6383515727,3.9785201431
C,2.0671105344,-1.0429219821,2.2396392923
C,0.2212359718,0.5350630175,1.8361631363
C,-0.2053265023,0.2543775856,3.1428493021
C,1.6163663295,-1.291026986,3.5238198927
H,-1.0911541771,0.7646958332,3.5062343816
H,2.1534393844,-1.9847610695,4.1611587253
H,0.092726677,-0.8197103955,4.9813083714
C,2.4736303411,0.4515507526,0.1507449459
C,0.6012006723,0.7753954131,-1.9350524247

C,2.2868233801,1.74430076,-0.4671983379
C,1.6760569333,-0.6617688967,-0.3106341686
C,0.6804179756,-0.4726227318,-1.3520548418
C,1.4079260774,1.8708707874,-1.5055018392
H,2.9065420004,2.5720812749,-0.142635445
H,1.3068120508,2.8219735435,-2.0175723212
H,-0.0983402071,0.9462765236,-2.7457238923
H,3.4486334684,0.2642776874,0.5845692701
H,2.0487518185,-1.6688518081,-0.1774765391
C,-0.1287042366,-1.6278337287,-1.7907187228
C,-1.6947056035,-3.8276874888,-2.6017060734
C,-0.6188868864,-1.7169924156,-3.1089066816
C,-0.4311177952,-2.6691906894,-0.8916916008
C,-1.2096539561,-3.7558185304,-1.2930691083
C,-1.393519931,-2.8048681257,-3.5079960646
H,-0.3728095774,-0.949068994,-3.8350513918
H,-0.0797776937,-2.6244226204,0.1345311984
H,-1.439406226,-4.5426964796,-0.5812291172
H,-1.7538385757,-2.8595958213,-4.5306592969
H,-2.2972106965,-4.6746705861,-2.9151476585
C,-0.5906424729,1.5440457841,1.0880278261
C,-2.2172780873,3.4718160492,-0.1448968234
C,-0.2859351675,2.9089454517,1.204522039
C,-1.7318000235,1.1544846126,0.3722897311
C,-2.5374246063,2.1147404293,-0.2459593388
C,-1.0936159628,3.8670857078,0.5873567321
H,0.5795725259,3.221573764,1.7813779896
H,-1.9933139572,0.103222669,0.3009147405
H,-3.4188049511,1.8010747477,-0.7971802235
H,-0.8491281342,4.9203785179,0.6869075793
H,-2.8466995184,4.2171226513,-0.6217627618
H,2.9566647556,-1.5516368406,1.8815591415
Imaginary Mode = -265.0411

TS18

Charge = 1 Multiplicity = 1
C,0.4860333248,1.1727074915,0.7383544169
C,-0.6678995164,2.0290950602,3.129656674
C,0.6687269923,0.3889061623,1.9156837871
C,-0.2691369223,2.3644367211,0.7784601534
C,-0.8387687364,2.7910029705,1.9653184114
C,0.0759077305,0.8488392604,3.0980609572
H,-0.4055600023,2.950542314,-0.1225293155
H,-1.4154823564,3.7091255519,1.9867389031
H,0.2081315611,0.2675762222,4.0044281931
H,-1.1150536079,2.3562799715,4.0628520759
C,1.659113699,0.9657433038,-0.4986103352
C,-0.0465759065,1.7828569883,-2.589236961
C,2.0063644765,2.1108646237,-1.3145367691
C,0.4428771491,0.2438722913,-0.7845915194

C,-0.4396640349,0.6861552661,-1.8528695061
C,1.173772053,2.486494644,-2.3282017229
H,2.9431984275,2.6221230777,-1.1250917061
H,1.4352164204,3.3243033256,-2.9663897229
H,-0.6724850107,2.1328014652,-3.4031211258
H,2.4702670923,0.4378102849,-0.0106131012
H,0.3561026871,-0.7814289341,-0.4451639382
C,-1.6746117658,-0.0739031203,-2.1367201232
C,-4.0466914147,-1.4991923526,-2.6643633955
C,-2.3850214239,-0.6997550039,-1.0947180276
C,-2.1714384374,-0.1826157874,-3.4503310783
C,-3.3456005193,-0.8879866864,-3.7098020783
C,-3.5623266512,-1.4022165269,-1.3569562448
H,-2.035792187,-0.6170541843,-0.0703237157
H,-1.6239958509,0.2609584319,-4.2760352627
H,-3.7076376993,-0.97005554,-4.7300528418
H,-4.1021531801,-1.8690306722,-0.538790492
H,-4.9597439254,-2.0498795669,-2.8686397291
C,1.4620062963,-0.8783343541,1.9553716295
C,2.9451816448,-3.2587911271,2.1167924249
C,2.8510386404,-0.8381700046,2.1637663283
C,0.8217462615,-2.1233908932,1.8448389418
C,1.5622400635,-3.3063966059,1.92166517
C,3.5869555172,-2.0231687852,2.2423063958
H,3.3531935192,0.1182313343,2.2809237787
H,-0.2551494633,-2.1694629861,1.7105090725
H,1.0554422001,-4.2625870091,1.83481047
H,4.6589340951,-1.9786059941,2.4082709606
H,3.5183396604,-4.1787007776,2.1788798283

Imaginary Mode = -201.8372
